# Path analysis of biomarkers for cognitive decline in early Parkinson’s disease

**DOI:** 10.1371/journal.pone.0268379

**Published:** 2022-05-13

**Authors:** Dmitri K. Gramotnev, Galina Gramotnev, Alexandra Gramotnev, Mathew J. Summers

**Affiliations:** 1 Research and Data Analysis Centre, Brisbane, Queensland, Australia; 2 Sunshine Coast Mind & Neuroscience – Thompson Institute, University of the Sunshine Coast, Birtinya, Queensland, Australia; 3 School of Health and Behavioural Science, University of the Sunshine Coast, Sippy Downs, Queensland, Australia; Cardiff University, UNITED KINGDOM

## Abstract

Clinical and biochemical diversity of Parkinson’s disease (PD) and numerous demographic, clinical, and pathological measures influencing cognitive function and its decline in PD create problems with the determination of effects of individual measures on cognition in PD. This is particularly the case where these measures significantly interrelate with each other producing intricate networks of direct and indirect effects on cognition. Here, we use generalized structural equation modelling (GSEM) to identify and characterize significant paths for direct and indirect effects of 14 baseline measures on global cognition in PD at baseline and at 4 years later. We consider 269 drug-naïve participants from the Parkinson’s Progression Marker Initiative database, diagnosed with idiopathic PD and observed for at least 4 years after baseline. Two GSEM networks are derived, highlighting the possibility of at least two different molecular pathways or two different PD sub-types, with either CSF p-tau181 or amyloid beta (1–42) being the primary protein variables potentially driving progression of cognitive decline. The models provide insights into the interrelations between the 14 baseline variables, and determined their total effects on cognition in early PD. High CSF amyloid concentrations (> 500 pg/ml) are associated with nearly full protection against cognitive decline in early PD in the whole range of baseline age between 40 and 80 years, and irrespectively of whether p-tau181 or amyloid beta (1–42) are considered as the primary protein variables. The total effect of depression on cognition is shown to be strongly amplified by PD, but not at the time of diagnosis or at prodromal stages. CSF p-tau181 protein could not be a reliable indicator of cognitive decline because of its significantly heterogeneous effects on cognition. The outcomes will enable better understanding of the roles of the clinical and pathological measures and their mutual effects on cognition in early PD.

## 1 Introduction

Deterioration of cognitive functions is a common and one of the most debilitating symptomatic manifestations of Parkinson’s disease (PD), causing detrimental impacts on the patients’ quality of life [1], with about 80% of all PD diagnosed patients eventually progressing to cognitive impairment and dementia [2, 3]. At the same time, the significant heterogeneity of this disease and its progression [4–6] impedes prediction of the onset and rate of cognitive decline in individual PD patients.

Various measures are associated with cognition in early PD, including: age [7], education [7], gender [8, 9], olfaction [8, 10–12], REM behavior disorder [13], motor impairment [9, 14], striatum dopamine transporter (DaT) imaging measures [15, 16], depression [17], and certain blood [18–22] and cerebrospinal fluid (CSF) measures [20, 21, 23–25]. However, so far, none of the known individual pathological or clinical measures has been accepted as an efficient or reliable biomarker for cognitive decline during PD progression [24, 26, 27]. It could be contended that no individual measure, clinical or pathological, is able to adequately reflect and incorporate the significant heterogeneity of PD and its progression, including in the cognitive domain. For example, although DaT imaging was recently indicated by the European Medicines Agency and Food and Drug Administration as an ‘enrichment biomarker’ for inclusion in clinical trials [28], four significant reservations and lack of reliability of this biomarker (when it comes to prediction of PD progression) have also been highlighted [29].

In an attempt to resolve this difficulty, optimized combinations of clinical and pathological measures (the integrated biomarkers) were used at baseline to predict the likely rate of cognitive decline in early PD patients [30–32]. The approach to the construction of the integrated biomarkers and the associated clinical scores proposed by [30–32] was based on the regression modelling involving multiple predictor variables. Although the developed models did consider possible interactions between the considered predictor variables [32], it was still unclear whether or not there were any significant mutual effects between the multiple predictor variables in the combinations constituting the integrated biomarkers. This is an important question, as the presence of such mutual effects between multiple predictor variables could cause difficulties for multiple regression models and obscure the total effects and importance of the individual variables for prediction of cognitive function and its decline in PD patients. For example, if two or more predictor variables significantly correlate with each other, the significance of their effects on cognition in the multiple regression models could be suppressed [33]. This could happen not because of the lack of significance of the suppressed variables in predicting cognition, but because they are also related with other predictor variables, and their predictive relationship with cognition is being masked. This could cause potentially incorrect or exaggerated/biased outcomes [33]. This issue could partially be dealt with by using the model averaging approach to computationally identify the most important predictor variables [32]. However, this still does not allow the accurate determination of the total effects of predictor variables on cognition, as some of these variables could influence cognition through mediation of other predictor variables. For example, age could have an effect on the levels of total tau (t-tau) in CSF, whereas t-tau might have an effect on cognition. Therefore, there could be an indirect effect of age on cognition through the mediation of t-tau, and this effect would be in addition to any direct effect of age on cognition (thus providing an additional insight into the mechanisms of the effects of age on cognition). It could be argued that path analysis [34] should offer an excellent opportunity to develop a useful insight into the mechanisms of cognitive decline in PD, and to properly take into account possible mutual relations between the predictor variables and determine the total effects of these variables on cognition.

The four CSF measures commonly evaluated in PD patients include alpha synuclein (α-syn), total tau (t-tau), phosphorylated tau 181 (p-tau), and amyloid beta 1–42 (Aβ_1–42_) [25]. The key roles of, and possible synergy between, these proteins in a variety of neurodegenerative diseases including PD were indicated [21, 35–37]. Previous studies have discussed in detail the diversity of PD manifestation and pathology [5, 35]. These studies include the interrelation between, and co-contributions from, the two major pathologies: synucleinopathy (i.e., classic PD α-syn cortical pathology) and neutritic amyloidopathy (i.e., Alzheimer pathology) [5], and also the particular contribution of cortical burden of tau neurofibrillary tangles [35, 36]. Misfolded tau, amyloid beta and α-syn could all possess prion properties, causing further misfolding of each other and propagation of the pathological patterns throughout the brain [37–39].

Currently, there is no consensus as to a primary biochemical cause for the developing PD pathology, although misfolded α-syn was argued as a potential key protein and driving force behind the pathogenic processes in PD [37]. Similarly, amyloid peptides and tau proteins have been suggested as the key factors for Alzheimer’s pathology [5, 35, 36, 38, 40, 41] that is a common comorbid pathology in PD [5, 35–37]. As a result, any further evidence elucidating potentially causal relations between the CSF measures of α-syn, t-tau, p-tau, and Aβ_1–42_, particularly in early PD, as well as their association with cognitive decline, is essential for better understanding of the underlining biochemical processes and biomarkers for PD progression. Path analysis should be one of the important statistical tools to provide such potential indications of possible causalities between the indicated CSF measures and their relationships to cognitive decline in PD.

The aim of this study is to apply the generalized structural equation model (GSEM) [34] to conduct comprehensive path analysis for prediction of global cognition in PD patients at baseline and 4 years later, on the basis of 14 baseline clinical and pathological measures. This analysis will identify and characterize a set of baseline variables capable of predicting the global cognition function. Significant non-linear effects (including indirect effects) of baseline α-syn, Aβ_1–42_, and age on global cognition will also be demonstrated and characterized. The outcomes will include the development of comprehensive networks of significant direct and indirect effects (effect paths) for the considered baseline variables on cognition in PD patients, thus offering important insights into the mechanisms of impacts of the these baseline variables on PD progression. In particular, two different networks of mutual effects between the measured CSF biochemical parameters will be proposed and justified, indicating possible causalities between these important measures. Discussion and interpretation of the outcomes will also be presented in the light of the currently existing literature findings and hypotheses.

## 2 Participants and methods

### 2.1 Study participants

The data used for this study was downloaded from the Parkinson’s Progression Markers Initiative (PPMI) database on January 4, 2018, according to the guidelines for data access and use. The PPMI initiative is an international, multi-site, longitudinal study of PD funded by the Michael J. Fox Foundation and partners [42]. The PPMI study and protocols was approved by the institutional review boards at the 24 enrolment sites, including the provision of written informed consent to participate from all participants [9, 25]. The ClinicalTrials.gov identifier for the PPMI study is NCT01141023. The approval for the use of the data in the current study was given by PPMI and Michael J. Fox Foundation.

The current study considered 269 patients from the PPMI database, who satisfied the following inclusion criteria: (1) all participants had been diagnosed with idiopathic PD within two years prior to the initial screening visit and record of their baseline characteristics in the PPMI database [25]; (2) the period of observation of each participant after the initial visit was no less than 48 months; (3) none of the participants were treated for PD prior to recording their baseline characteristics in the PPMI database; (4) all participants displayed dopamine transporter deficit on the DaT scan at baseline, which was determined using the dopamine transporter single-photon emission computer tomography (DaT) averaged over the striatum [43–45]; (5) participants had asymmetric resting tremor or asymmetric bradykinesia, or at least two of the following: resting tremor, bradykinesia, and rigidity; (6) all participants had mild to moderate disease severity (Hoehn and Yahr stages I or II [46]) at baseline; and (7) participant’s age was ≥ 30 years.

### 2.2 Variables

The cognitive function of the study participants at baseline and 4 years later (in months 48–51) was evaluated using the Montreal Cognitive Assessment (MoCA) scale that was a widely validated and used instrument for the evaluation of global cognitive functions, including in PD [25, 30, 31, 47, 48]. Total MoCA scores were calculated for each participant. It was assumed that MoCA scores at 4 years after baseline (MoCA_4y_) were dependent on the MoCA scores at baseline (MoCA_b_).

Fourteen other demographic, clinical, and pathological measures evaluated at baseline were also considered, including: age, years of prior education, gender, University of Pennsylvania Smell Identification Test score, baseline combined score for Sections 1, 2 and 3 of the Movement Disorder Society Unified Parkinson’s Disease Rating Scale (UPDRS_1–3_), rapid eye movement sleep behavior disorder score (RBD), Geriatric Depression Scale score (GDS), total State-Trait Anxiety Inventory score, DaT variable [43, 45] calculated as the average specific binding ratio over the dorsal striatum including the caudate nucleus and putamen, plasma levels of insulin-like growth factor 1, and four CSF measures: alpha synuclein (α-syn), total tau (t-tau), phosphorylated tau 181 (p-tau), and amyloid beta 1–42 (Aβ_1–42_). The collection process for the CSF measures was described elsewhere [25]. Summary statistics describing these 14 variables was presented in Supplementary Table 1 in [32].

**Table 1:** Direct effects for the GSEM network in Fig 1A.

Response Variable	Predictor Variable	Coefficient	*p*-value
**MoCA**_**4y**_	MoCA_b_	0.549	< 0.001
	UPDRS_1-3_	−0.0497	0.002
	Age	−0.462	0.005
	GDS	−0.202	0.031
	ln(α-syn)	−332.49	0.027
	ln^2^(α-syn)	45.172	0.026
	ln^3^(α-syn)	−2.0306	0.026
	ln(t-tau)	−2.769	0.001
	(Aβ_1–42_)^1/2^	12.010	0.020
	Aβ_1–42_	−0.60407	0.028
	(Aβ_1–42_)^3/2^	0.01015	0.034
	(Aβ_1–42_)^1/2^ × Age	0.0192	0.020
	constant	754.14	0.042
**MoCA**_**b**_	UPDRS_1-3_	−0.0248	0.038
	Education	0.1393	0.011
	Age	−0.0572	0.043
	Age^2^	0.00808	0.017
	Age^3^	−0.0000291	0.74
	Age^4^	−0.0000180	0.006
	Gender (base: Male)	0.631	0.063
	constant	25.47	< 0.001
**UPDRS**_**1-3**_	RBD	1.2098	< 0.001
	DaT	−8.932	< 0.001
	GDS	1.230	< 0.001
	Age	0.1655	0.031
	constant	36.45	< 0.001
**RBD**	(Aβ_1–42_)^1/2^	−0.173	0.005
	DaT	−1.03	0.016
	GDS	0.169	0.015
	constant	8.460	< 0.001

**Fig 1:**
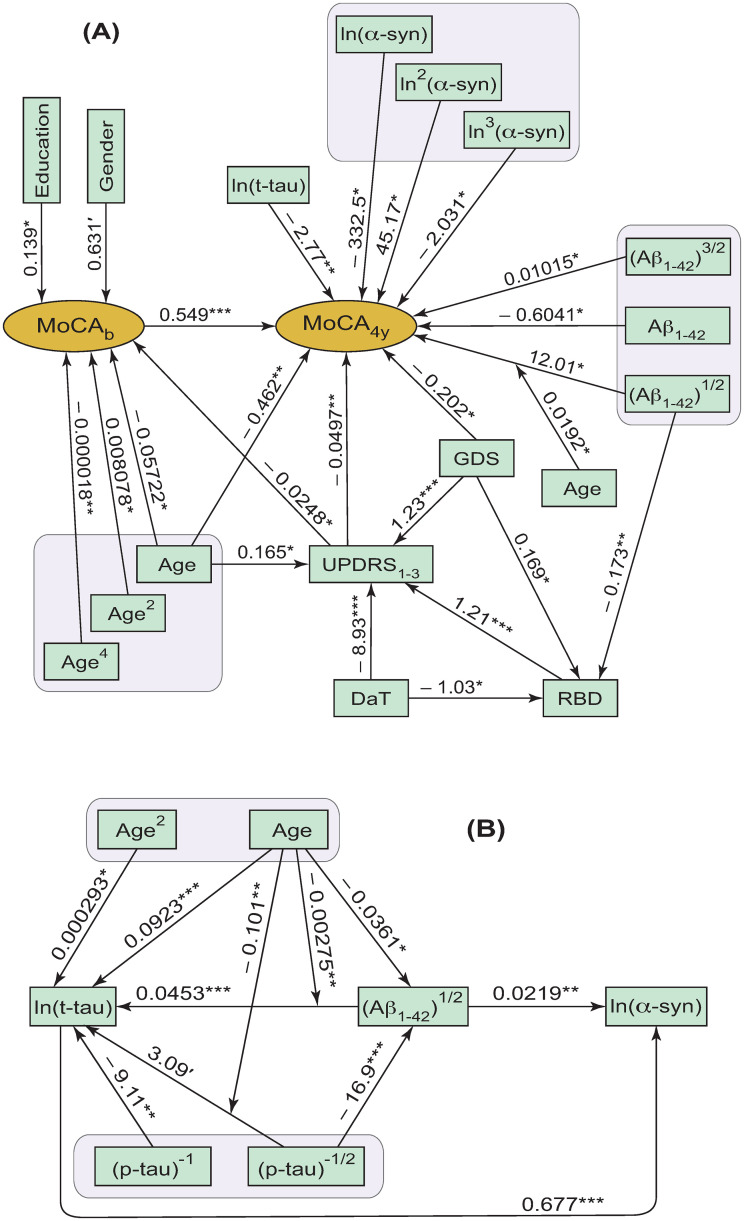
GSEM networks of significant (*p* < 0.1) direct and indirect effects for the fourteen baseline variables, MoCA_b_ and MoCA_4y_. **(A)** The network of effects on the MoCA scores at baseline and 4 years later; and **(B)** the additional network of effects between the CSF parameters and baseline age, with p-tau as the primary independent protein variable. The arrows between the variables show the directions of the direct effects. The arrows pointing to other arrows indicate the significant interaction effects. The asterisks indicate the levels of statistical significance of the respective regression coefficients: (***) *p* < 0.001; (**) 0.001 ≤ *p* < 0.01; (*) 0.01 ≤ *p* < 0.05; (′) 0.05 ≤ *p* < 0.1.

The outcomes of the GSEM model in Fig 1A, including the regression coefficients for the significant direct effects and their respective *p*-values. The numbers of decimal places in the coefficients are given to ensure < 1% errors in the respective effects on the endogenous variables.

Three other variables available from the PPMI database were the epidermal growth factor, triglycerides, and cholesterols. However, due to their limited sample size of 123 (Table 1 in S1 Text in [32]), these three variables were not included in the current study.

Sixty years (the average age of participants at baseline) was regarded as the origin for the age variable. This was done to obtain more meaningful regression coefficients for age, as there were no participants of age close to zero. In this case, the regression coefficients for the linear terms of age in the GSEM models could be interpreted as the rates of changing the variables affected by baseline age at 60 years.

### 2.3 Statistical methodology

The analysis was conducted using the Stata16 software package [49]. As explained above, the major goal of this study was to undertake comprehensive path analysis and modelling of the considered variables, with the intention to identify and characterize networks of their significant direct and indirect effects on the global cognition in PD at baseline and 4 years later (MoCA_b_ and MoCA_4y_, respectively). The statistical approach was based on the structural equation modelling (SEM) and generalized structural equation modelling (GSEM) [34].

The accepted level of statistical significance in this study was characterized by *p*-values below 0.1 (under 10% significance), although only two effects with *p* ≥ 0.05 were found in the developed models. No direct effects with *p* ≥ 0.1 are shown in the developed GSEM networks of significant effects (see Section 3.1 below).

The choice of SEM and GSEM as the statistical methodology for this study was dictated by the fact that many of the considered predictor variables were characterized by significant correlations (co-linearity) with each other. For example, UPDRS_1-3_ was significantly correlated with GDS, RBD, DaT, Age, Aβ_1–42_, and MoCA_b_. Consideration of all these mutually related variables cannot be fully justified in a single multiple regression with MoCA_4y_ being considered as the dependent variable [34]. Therefore, use of SEM and GSEM was essential to correctly take into account any possible mutual effects of the considered predictor variables on each other [34]. This methodology allowed the establishment and characterization of effect paths involving direct and indirect effects of the mutually related variables on MoCA_b_ and MoCA_4y_.

It was found that the distributions of the MoCA scores at baseline and 4 years later were censored from the right (Fig 1 in S1 Text). The analysis and modelling of such data, with the MoCA scores considered as the dependent variables, required the use of censored regressions [50]. This was one of the reasons for choosing GSEM as the main methodological tool for this study, because, unlike SEM, it allowed the use of censored regressions for path analysis [34]. In addition, unlike SEM, GSEM also allows systematic consideration of interactions between the predictor variables and any possible non-linearities of the effects of the predictor variables [34]. Nonetheless, SEM and its modification indices [34] were also used as an additional tool for the derivation and justification of the networks of direct and indirect effects involving the four CSF protein variables including α-syn, Aβ_1–42_, t-tau, and p-tau (S1 Text).

As per the standard requirement for model development, all numerical variables having significant effects on other variables were tested for any significant non-linear effects (including on the MoCA scores at baseline and 4 years later). Any significant non-linear terms were included in the final models. Possible interactions between the predictor variables were also considered and included in the final model, where significant.

The distributions of any endogenous variables (i.e., the variables significantly depending on other considered variables) were checked for normality, and the Tukey Ladder of Transformations [51] were used to determine the required variable transformations to achieve normality. In particular, variables t-tau, p-tau, Aβ_1–42_, and α-syn had to be transformed as: t-tau → ln(t-tau), *p* = 0.253; p-tau → (p-tau)^-1/2^, *p* = 0.152; Aβ_1–42_ → (Aβ_1–42_)^1/2^, *p* = 0.342; and α-syn → ln(α-syn), *p* = 0.057. As required for achieving normality of the transformed variables [51], each of these *p*-values was larger than the conventional threshold value of 0.05. The developed GSEM models incorporated the transformed versions of these four variables. Further description of the specific steps leading to the development of the GSEM models is presented in S1 Text. Graphic representation for the total effects was used to facilitate perception of the significant non-linear effects of age and the three CSF measures including p-tau, Aβ_1–42_, and α-syn.

## 3 Results and discussions

### 3.1 GSEM networks

**Fig 2:**
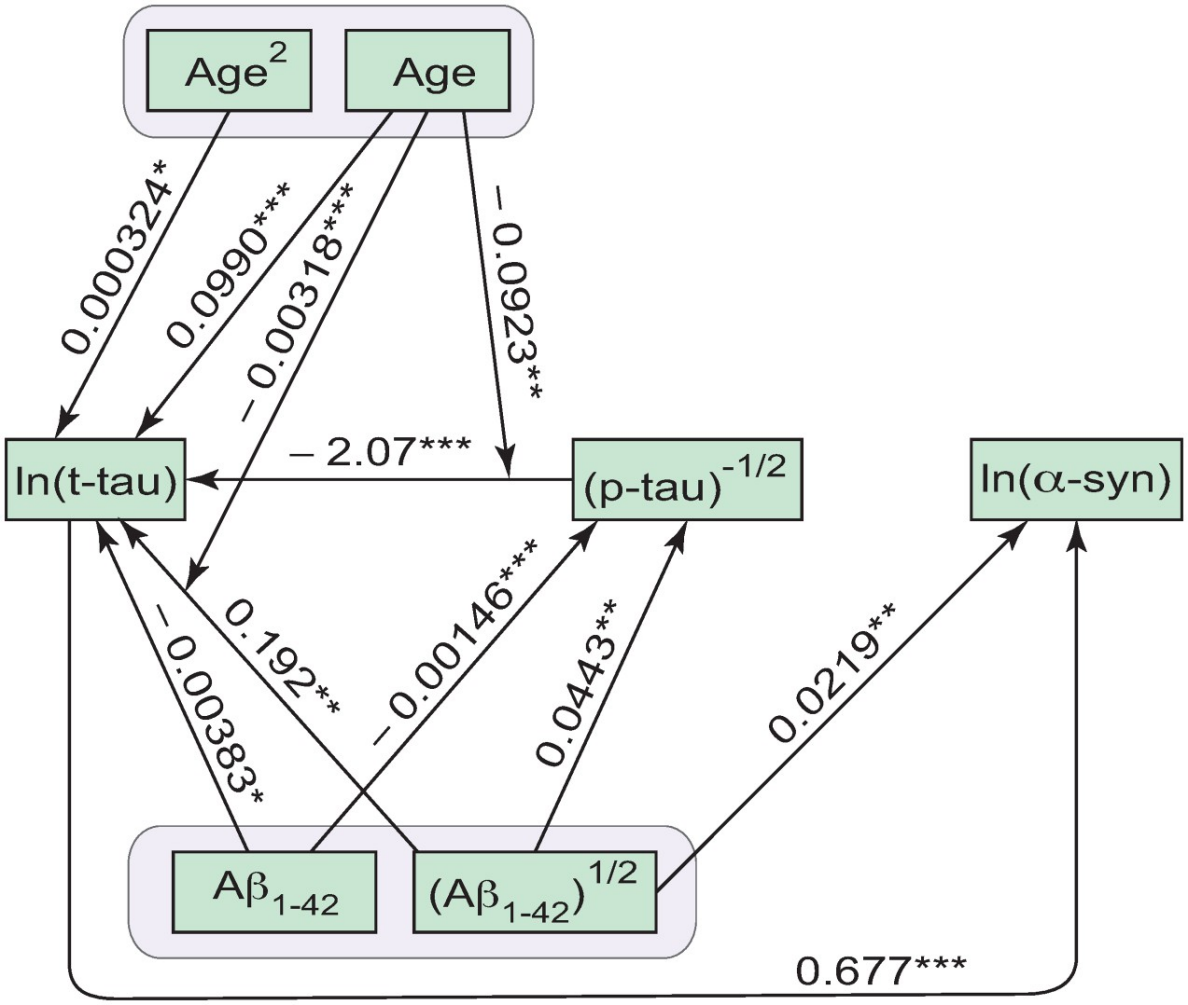
The alternative (to Fig 1B) network of effects between the four CSF measures and Age, with Aβ_1–42_ as the primary independent protein variable. The arrows between the variables show the directions of the direct effects. The arrows pointing to other arrows indicate the significant interaction effects. The asterisks indicate the levels of statistical significance of the respective regression coefficients: (***) *p* < 0.001; (**) 0.001 ≤ *p* < 0.01; (*) 0.01 ≤ *p* < 0.05.

As explained in more detail in S1 Text, the conducted GSEM analysis and justification of the networks of effects between the 14 baseline demographic, clinical, and pathological measures resulted in the two alternative models for prediction of global cognition scores MoCA_b_ and MoCA_4y_. One of these models is given by Fig 1A and 1B, and the other is given by Figs 1A and 2 (see also Tables 1 and 2). Note that Figs 1B and 2 are the two alternative extensions of Fig 1A, showing the additional relationships between the four baseline CSF measures and baseline age. Figs 1B and 2 are presented separately from Fig 1A to ease visual perception of the GSEM models and to highlight the relationships between the four CSF protein variables. The University of Pennsylvania Smell Identification Test score, total State-Trait Anxiety Inventory score, and plasma levels of insulin-like growth factor 1 are missing from Figs 1A, 1B and 2 because these variables did not have significant effects on MoCA or other variables.

**Table 2:** Direct effects for the GSEM Networks in Fig 1B (p-tau Model) and Fig 2 (Amyloid Model).

Response Variable		Predictor Variable	p-tau Model		Amyloid Model	
			Coefficient	*p*-value	Coefficient	*p*-value
**CSF Variables**	**ln(α-syn)**	(Aβ_1–42_)^1/2^	0.0219	0.006	0.0219	0.006
		ln(t-tau)	0.6773	< 0.001	0.6773	< 0.001
		constant	4.505	< 0.001	4.505	< 0.001
	**(Aβ**_**1–42**_ **)** ^**1/2**^	(p-tau)^-1/2^	−16.91	< 0.001	-	-
		Age	−0.0361	0.017	-	-
		constant	23.82	< 0.001	-	-
	**(p-tau)** ^**-1/2**^	(Aβ_1–42_)^1/2^	-	-	0.0443	0.001
		Aβ_1–42_	-	-	−0.00146	< 0.001
		constant	-	-	-	0.85
	**ln(t-tau)**	(p-tau)^-1/2^	3.0912	0.076	−2.068	< 0.001
		(p-tau)^-1^	−9.114	0.003	-	-
		(Aβ_1–42_)^1/2^	0.0453	< 0.001	0.192	0.006
		Aβ_1–42_	-	-	−0.00383	0.034
		Age	0.0923	< 0.001	0.0990	< 0.001
		Age^2^	0.0002936	0.031	0.000324	0.019
		(p-tau)^-1/2^ × Age	−0.1014	0.002	−0.0923	0.005
		(Aβ_1–42_)^1/2^ × Age	−0.00275	0.001	−0.00318	< 0.001
		constant	2.689	< 0.001	1.993	0.003

The outcomes of the two GSEM models in Figs 1B and 2, including the regression coefficients for the significant direct effects and their respective *p*-values. The p-tau Model is the model with (p-tau)^-1/2^ as the primary predicting protein variable (Fig 1B), and the Amyloid Model is the model with (Aβ_1–42_)^1/2^ as the primary predicting protein variable (Fig 2). The numbers of decimal places in the coefficients are given to ensure < 1% errors in the respective effects on the endogenous variables.

An overall conclusion from the developed models is that there are multiple baseline variables influencing global cognition of PD patients at baseline and 4 years later. These effects on cognition are direct and indirect for many of the involved variables. For example, UPDRS_1-3_ has direct effects on the MoCA scores at baseline and at 4 years later (Fig 1A). At the same time, UPDRS_1-3_ also has a significant indirect effect on MoCA_4y_ through the mediation of MoCA_b_ (Fig 1A). These direct and indirect effects should be added together to obtain the total effect of UPDRS_1-3_ on MoCA_4y_. The lists of the indirect effects on MoCA_b_ and MoCA_4y_, resulting from the developed models (Figs 1A, 1B and 2), are presented in Section 3 in S1 Text.

There were no significant direct effects of the CSF measures on MoCA_b_ (Fig 1A). The direct effects of the CSF measures on MoCA_4y_ and RBD are shown in Fig 1A. It could thus be argued that significant direct effects of baseline CSF measures on global cognition in PD could typically be observed as the disease progresses, but not at baseline. This, however, does not mean that the CSF measures do not have any effect on MoCA_b_. For example, Aβ_1–42_ has an indirect effect on MoCA_b_ through the mediation of RBD and UPDRS_1-3_ (Fig 1A). Because (p-tau)^-1/2^ has a significant direct effect on (Aβ_1–42_)^1/2^ (Fig 1B), it also has an indirect effect on MoCA_b_ (see Section 3 in S1 Text for the list of indirect effects on MoCA_b_ in the model given by Fig 1A and 1B).

In addition, the lack of significant direct effects of the four baseline CSF measures on MoCA_b_ (Fig 1A) could be a result of the sample size limitations. A larger sample could see some of these direct effects becoming significant. Therefore, replication of the obtained outcomes with different and/or larger samples of patients with early PD will be beneficial for the establishment of all significant measures predicting cognition at baseline and later into the disease progression. At the same time, as indicated in the previous paragraph, the current analysis suggests that the direct effects of the considered baseline CSF measures on global cognition at baseline are at least much smaller than the effects of the baseline CSF measures on MoCA_4y_.

The direct effects on MoCA_4y_ from the transformed ln(α-syn) and (Aβ_1–42_)^1/2^ variables are significantly non-linear, with the need to consider three powers of these transformed variables (Fig 1A). Disregarding these non-linear terms of the ln(α-syn) and (Aβ_1–42_)^1/2^ variables would have led to prediction errors and loss of significant non-linearities of the respective dependences of MoCA_4y_ on these variables. For better clarity, these two direct non-linear effects on MoCA_4y_ can be written as:

$$\begin{array}{cc} {\text{MoCA}}_{4\text{y}} & =k_{\alpha -\text{syn}1}\text{ln}(\alpha -\text{syn})\;+k_{\alpha -\text{syn2}}{\text{ln}}^{2}(\alpha -\text{syn})\;+k_{\alpha -\text{syn}3}{\text{ln}}^{3}(\alpha -\text{syn})\;+\\ & +k_{\text{A}\beta 1}{\left({\text{A}\beta }_{1-42}\right)}^{1/2}+k_{\text{A}\beta 2}\left({\text{A}\beta }_{1-42}\right)\;+k_{\text{A}\beta 3}{\left({\text{A}\beta }_{1-42}\right)}^{3/2}+\;\ldots , \end{array}$$ (1)

where the coefficients in front of the powers of ln(α-syn) and (Aβ_1–42_)^1/2^ are those shown in Fig 1A, respectively, and the symbol ‘…’ indicates any additional terms representing the significant direct effects on MoCA_4y_ from such variables as ln(t-tau), Age, UPDRS_1-3_, GDS, and MoCA_b_ (Fig 1A).

Multiple earlier studies suggested the existence of a possible link between PD progression in the form of cognitive decline and the presence of Alzheimer’s comorbid pathology [5, 35–37]. Therefore, the determination of potential causalities between different proteins and peptides in the brain and CSF could be a key for the detailed understanding of the driving biochemical forces behind PD progression, including stratification of PD subtypes, prediction of serious cognitive complications, and development and application of more targeted treatments. Figs 1B and 2 present the derived networks of potentially or partially causal effects between the four CSF measures and age of the participants.

We use the term ‘potentially or partially causal effects’ because the developed GSEM models and the way that they were derived (Section 2 in S1 Text) provide only reasonable indications of causalities, rather than their definitive proof. These indications are based on the conducted optimization of the models (including the directions of any direct effects) to ensure the best possible fit of the models to the available data. Therefore, the available outcomes could, to a certain degree, be cohort-specific. The term ‘partially causal’ reflects the possibility that not all, but only part of, variance of one variable could be causally dependent on part of variance of another variable. Further validation of the indicated causalities and developed networks should be based on the similar derivation and comparison of optimal effect networks resulting from different cohorts of participants with early PD.

The first of the developed networks of effects (Fig 1B) identifies p-tau as the primary independent CSF protein variable, that is, as the potential driving force, or a trigger, for the pathological changes in the other proteins. This is because, according to the developed model (Fig 1B), the CSF levels of p-tau are not affected by the other three CSF proteins including Aβ_1–42_, t-tau, or α-syn. On the other hand, CSF levels of p-tau appear to have direct or indirect effects on CSF levels of t-tau, Aβ_1–42_, and α-syn (Fig 1B), which, in turn, have significant effects on the global cognition measured by MoCA_4y_ at 4 years after baseline (Fig 1A). In this sense, CSF p-tau works as a predictor for the other three CSF proteins and global cognition in PD patients. The alternative network (Fig 2) identifies Aβ_1–42_ as the primary independent CSF protein variable predicting the other three CSF proteins (t-tau, p-tau, and α-syn).

The relationships between the CSF concentrations of the considered proteins and progression of PD pathologies and symptoms could be rather complex. For example, CSF levels of all the four proteins at baseline (i.e., at early stages of PD) could be lower in PD patients compared to controls [21, 23–25, 52, 53], but increase at later stages of the disease [54]. It was suggested that CSF levels of p-tau increasing over 2 years could be associated with faster cognitive decline [54], whereas other studies suggested that lower baseline concentrations of Aβ_1–42_ in CSF were associated with more rapid cognitive decline in PD [20, 23, 25, 36, 55, 56]. In addition, significant correlations exist between the CSF levels of all considered four proteins [54], and this is consistent with the synergy of their associated pathologies suggested by the autopsy studies [5, 35–37].

The models developed in the current study demonstrate that either p-tau (Fig 1B), or Aβ_1–42_ (Fig 2) could be considered as the alternative biochemical triggers for progression of cognitive decline in early PD. Because of the better model fit (Table 2 in S1 Text), the model shown in Fig 1B could be preferable (compared to the model shown in Fig 2) for prediction of the global cognitive function in PD patients at early stages of the disease. Therefore, it can be hypothesized that cognitive decline in PD is more likely to arise from CSF p-tau proteins that predict (on average) the remaining three CSF measures including Aβ_1–42_, t-tau, and α-syn (Fig 1B). The somewhat less (but not much less–Table 2 in S1 Text) likely alternative is that CSF Aβ_1–42_ could be the origin/trigger of cognitive decline in PD, and predicts p-tau, t-tau, and α-syn (Fig 2). The fundamental difference between the two models (Figs 1B and 2) is not necessarily in the prevalence of amyloid or tau pathologies, but rather in whether the CSF levels of p-tau tend to predict the CSF amyloid beta (Fig 1B), or vise versa (Fig 2). In any case, both these alternative outcomes appear to be compatible with the findings that Alzheimer’s comorbid pathology in the form of amyloid beta depositions and tau neurofibrillary tangles (expectedly causing changes of CSF p-tau and Aβ_1–42_ levels) is associated with more rapid and significant cognitive decline [5, 35–37].

It is possible to hypothesize that the two alternative networks of effects between the four CSF measures and Age (Figs 1B and 2) could be related to two different sub-types of PD. In one of these sub-types, p-tau changes in CSF could be primary to the loss of cognitive function in PD progression, whereas for the second sub-type Aβ_1–42_ could work as a trigger for the loss of cognitive function. The better fit for the model with p-tau predicting the other proteins (Table 2 in S1 Text) could be a reflection of a greater prevalence of the p-tau subtype of PD (Fig 1A and 1B) in the available sample of participants. Alternatively, the two different models (Figs 1B and 2) could also be a reflection of the two different molecular paths towards cognitive decline in PD, both of which occurring in every PD patient, or some of them. In this case, part of variance of CSF p-tau is predicted by part of variance of CSF Aβ_1–42_, whereas some other part of variance of CSF p-tau predicts some other part of variance of CSF Aβ_1–42_ (potential causalities going both ways).

Both the models (Figs 1B and 2) are consistent with the finding of prion-like properties of misfolded p-tau and amyloids [38, 57]. The two networks in Figs 1B and 2 could be reflections of the two prion-like processes/paths originating from p-tau and Aβ_1–42_, respectively. These two processes/paths could occur together or with preferences to one of them.

Interestingly, p-tau does not have a significant direct effect on global cognition either at baseline, or 4 years later (Fig 1A). The effects of p-tau on cognition are only indirect through the mediation of the other CSF proteins (Figs 1B and 2). This is compatible with the comparatively low relative variable importance of p-tau in predicting the rate of cognitive decline in PD [32]. This could also explain the earlier propositions that the findings about phosphorylated tau as a biomarker of cognitive decline in PD are inconsistent [36, 58] (see also Section 3.4.3 below).

Because α-syn depends on all other CSF proteins and not the other way around (Figs 1B and 2), neither of the two developed networks suggested α-syn as the primary trigger for the protein pathology in PD. This is despite the wide acceptance that synucleinopathy (in the form of Lewy bodies and/or Lewy neurites) constitutes the hallmark of PD pathology [5, 37, 39, 59, 60]. The developed models (Figs 1B and 2) indicate a possibility that synucleinopathy could be secondary to the comorbid Alzheimer’s neuropathology involving amyloids and misfolded tau protein. This does not exclude the possibility of subsequent propagation of misfolded α-syn from cell to cell throughout the interconnected brain regions in a prion-like fashion [39, 57]. It is also possible that, once the pathological process has been initiated with the involvement of p-tau and/or Aβ_1–42_ (Figs 1B and 2), cross-fibrillization of tau and α-synuclein, involving different strains of pathological α-syn, could also occur [35, 39, 61], potentially resulting in different PD sub-types. These views and arguments are consistent with the synergetic nature of, and strong correlations between, α-syn neuropathology and Alzheimer’s disease neuropathology in PD [35, 36, 61, 62].

### 3.2 General comments about the models

It is necessary to keep in mind that the arguments and hypotheses presented in the previous section are based on the consideration of CSF levels of the considered four proteins, and not on their direct presence in the brain. Although it is expected that the obtained relationships between these CSF markers could largely be reflective of the processes in the brain, direct extensions of these relationships and potential causalities to the processes in the brain should be done with caution, as changes in the brain might not be equivalent at all times to changes in CSF. Direct analysis of protein effect networks in the brain in early PD may be difficult in humans, although it might be attempted in future animal models of PD. This might shed more light on the links between what is observed in CSF and what happens in the brain, including whether or not the developed CSF protein networks (Figs 1B and 2) are fully and adequately reflective of the brain processes.

Although comparisons between the studies focusing on CSF analysis at early PD stages and autopsy studies are important, they should be made with caution. This is because molecular findings at autopsy are often secondary to the disease process [63]. CSF findings at early stages of PD (which might be different from the ‘final stage’ autopsy findings) are more likely to shed light on possible biochemical causality for PD progression, including in the global cognitive domain.

The predicted networks of the effects between the four CSF measures and Age (Figs 1B and 2) are a reflection of the average trends for the considered cohort of PD patients. Vast heterogeneity of PD may result in a variety of specific progression courses [35, 36], including with potentially different outcomes with regard to cognitive dysfunction, or interrelations between the four CSF measures.

The amyloid hypothesis of Alzheimer’s disease assumes a cascade of events initiated by amyloid beta deposition followed by tau pathology, synaptic damage, inflammation response, and neurodegeneration [40, 41, 64]. On the other hand, as discussed in the previous section, it is currently understood that Alzheimer’s comorbid pathology associated with amyloid beta deposition and formation of tau tangles is responsible for more rapid and significant cognitive decline in PD [5, 35–37]. It could be hypothesized that the development of Alzheimer’s comorbid pathology in PD and the associated cognitive decline are also largely associated with the amyloid hypothesis. The developed models (Figs 1B and 2) identify CSF p-tau and Aβ_1–42_ as the primary protein triggers for progression of cognitive decline in PD–consistently with the expectation that the amyloid hypothesis for Alzheimer’s comorbid pathology should be associated with changes in CSF levels of these proteins at the early stages of PD. The second model (Fig 2) directly identifies CSF Aβ_1–42_ as the primary protein trigger of cognitive decline in PD, which agrees with the amyloid hypothesis for Alzheimer’s pathology [40, 41, 64]. The first model (Fig 1B) suggests that CSF Aβ_1–42_ levels are influenced by the levels of p-tau. Molecular or cellular mechanisms of such influence are not clear at this stage. As also indicated in the previous section, it might be possible that amyloid and tau pathologies are influencing each other to produce mutual causal effects in both directions, resulting in the two different networks for the CSF protein levels (Figs 1B and 2). This would be in line with the proposed synergy between α-syn, p-tau and Aβ_1–42_ in PD [21, 35–37]. Future analysis of patient cohorts at different stages of PD (including the prodromal stage) might be beneficial for shedding more light on this possible synergy and mutual effects involving the CSF protein variables.

Validations of the developed GSEM models (Figs 1A, 1B and 2 and Tables 1 and 2) were conducted using internal cross-validation on the basis of the bootstrapping procedure, and Bonferroni-type procedures for simultaneous testing of multiple hypotheses. For more detail about the methodologies and outcomes of these validations see Section 4 in S1 Text. As indicated above, cross-cohort validation of the developed models should be a matter for future research involving different compatible cohorts of participants.

### 3.3 Effects of baseline age on global cognition

**Fig 3:**
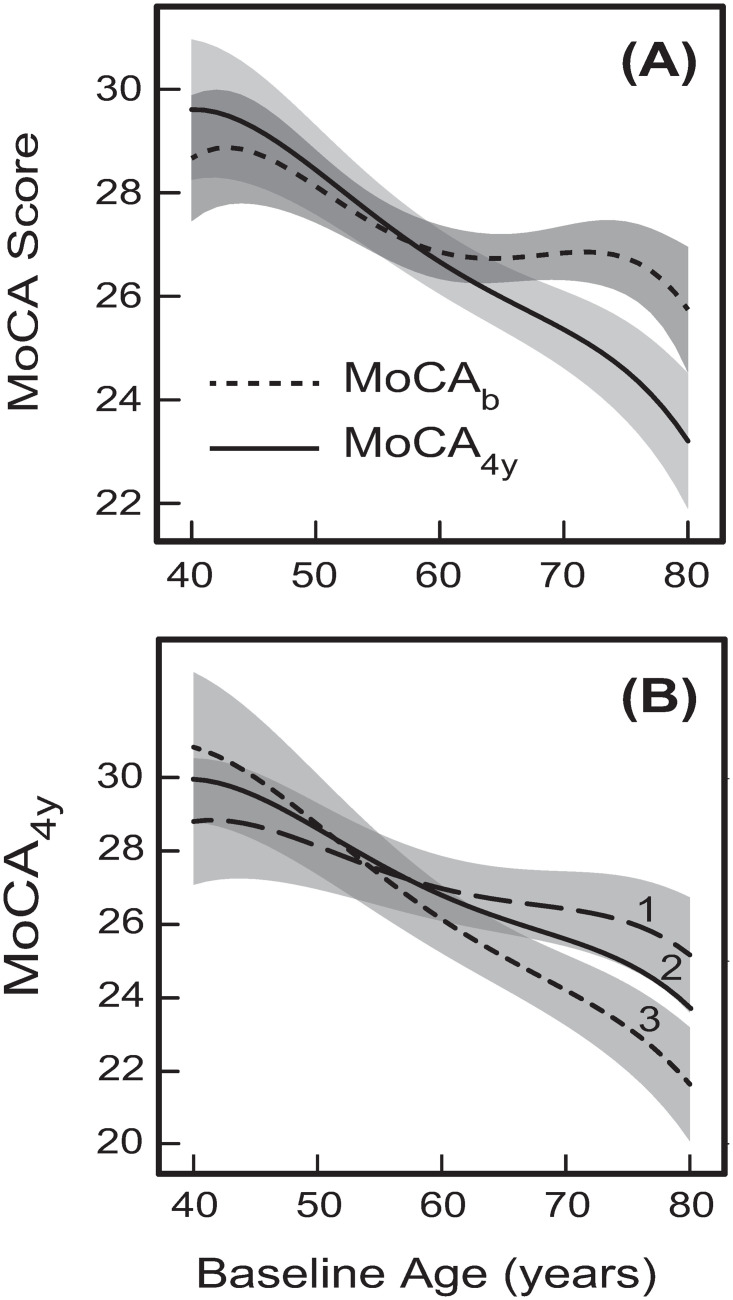
The predicted dependences of the MoCA scores on baseline age. **(A)** The age dependences for the model with CSF p-tau as the primary predicting protein variable (Fig 1A and 1B); and **(B)** the age dependences for the model with Aβ_1–42_ as the primary predicting protein variable (Figs 1A and 2). The three curves in **(B)** are for the three different values of CSF Aβ_1–42_ concentration of 494 pg/ml (0.9 percentile–curve 1), 364 pg/ml (0.5 percentile–curve 2), and 251 pg/ml (0.1 percentile–curve 3). The shaded bands indicate the 95% prediction intervals for the respective dependences. For ease of visual perception, the prediction intervals are not shown for curve 2 in **(B)**. The other variables were taken at their mean values or base categories: **(A)** DaT = 1.38, GDS = 2.30, Gender = Male, Education = 15.64 years, and average (p-tau)^-1/2^ = 0.283 (p-tau = 12.49 pg/ml); **(B)** DaT = 1.38, GDS = 2.30, Gender = Male, and Education = 15.64 years, or predicted according to the effect paths in the respective GSEM models (Figs 1A, 1B and 2).

The Age variable has a direct linear effect on MoCA_4y_ (Fig 1A). At the same time, Age also has a significant non-linear direct effect on MoCA_b_ (Fig 1A), whereas MoCA_b_ has a significant direct effect on MoCA_4y_ (Fig 1A). This means that Age has a significant non-linear indirect effect on MoCA_4y_ through mediation of MoCA_b_. In addition, in the model shown in Fig 1B, Age has a significant direct non-linear effect on ln(t-tau) and significant direct linear effect on (Aβ_1–42_)^1/2^, whereas ln(t-tau) and (Aβ_1–42_)^1/2^ have direct linear and non-linear effects, respectively, on MoCA_4y_ (Fig 1A). Therefore, Age has additional significant indirect non-linear effects on MoCA_4y_ through mediation of ln(t-tau) and (Aβ_1–42_)^1/2^. Further, in the model shown in Fig 1, Age has 8 other indirect effects (11 in total) on MoCA_4y_ through mediation of such variables as ln(t-tau), (Aβ_1–42_)^1/2^, ln(α-syn), MoCA_b_, RBD, and UPDRS_1-3_ (for the lists of the indirect effects see Section 3 in S1 Text). Thus, for the model shown in Fig 1, the total effect of Age on MoCA_4y_ will then be the sum of the direct linear effect and the 11 linear and non-linear indirect effects (plus any additional effects from the interaction terms between Age and any other variables). The predicted total dependence of MoCA_4y_ on baseline age (the total effect of baseline age on MoCA_4y_) is shown in Fig 3A by the solid curve. Similarly, the predicted total effect of baseline age on MoCA_b_ is also a non-linear function shown in Fig 3A by the dotted curve.

It is interesting that the solid curve in Fig 3A goes above the dotted curve for baseline age < 58 years. This suggests that patients with earlier onset of PD (< 58 years) are predicted to perform somewhat better in terms of their global cognition 4 years after baseline. However, the difference between the two curves at Age < 58 years (Fig 3A) is not statistically significant (with *p* > 0.15 even for Age = 40 years). In addition, the overall good cognitive performance and learning abilities at younger age, e.g., < 50 years, could further explain better (though not significantly better) performance of the younger participants 4 years after baseline. The younger participants with good cognitive function might have learned and remembered how to respond to the MoCA test that was typically administered multiple times over the 4-year period. This could have resulted in some bias of the subsequent MoCA tests (including the one at 4 years after baseline), causing the predicted increase in cognitive performance among younger participants with better global cognition (Fig 3A). Even further, the baseline MoCA test could have also been associated with a greater level of psychological discomfort, stress and negative expectations, causing some additional decline in the outcomes, whereas 4 years later, these discomfort, stress and negative expectations are likely to diminish (particularly where multiple MoCA tests were performed over this period of time). As a result, MoCA scores 4 years after baseline might be biased (though insignificantly–Fig 3A) towards higher values.

It is important to note that this potential bias is less likely to occur for older participants with compromised global cognition, memory, and learning abilities. In particular, it can be seen that, for baseline ages of > 64 years, MoCA_4y_ is predicted to be significantly lower than MoCA_b_ (*p* < 0.1)–Fig 3A. This is also consistent with the previous findings that patients with PD who are younger at the onset are more likely to survive longer without significant cognitive decline and dementia [35]. Less comorbid Alzheimer’s disease neuropathology typically occurs in PD patients with earlier onset [35], which is also consistent with more benign and slow PD progression.

One of the indications resulting from here is that the MoCA test should be used with caution when monitoring longitudinal variations in global cognition. Excessively frequent use of this test (e.g., several times per year) could cause accumulation of the bias towards better cognitive results among younger patients with initially good cognition and memory.

The dependence of MoCA_b_ on Age is practically the same for both the developed models (Figs 1A, 1B and 2). The discrepancies are only due to the differences in the indirect effects of Age on MoCA_b_ through mediation of amyloid beta, RBD, and UPDRS_1-3_ (Fig 1A). These indirect effects cause only small alterations in predictions of MoCA_b_ by Age, so that the resultant dependences are indistinguishable on the scale of Fig 3A. Therefore, we do not present the additional curve for MoCA_b_ as a function of Age in Fig 3B for the second GSEM model (Figs 1A and 2).

The comparison of curve 2 in Fig 3B and the solid curve in Fig 3A suggests that these curves are rather similar in terms of their shapes and the corresponding values of the MoCA_4y_ scores. This could be expected, as the difference between these two curves is again caused by the differences in the protein networks in Figs 1B and 2. These network differences are related only to the different indirect effects of Age on MoCA_4y_, and their contributions to the total effect of Age are rather small.

The differences between the three curves in Fig 3B illustrate the effects of different baseline CSF concentrations of Aβ_1–42_ on global cognition 4 years after baseline. In particular, increasing baseline CSF concentration of Aβ_1–42_ results in a significant increase of MoCA_4y_ for patients older than 65 years at baseline (Fig 3B). More detailed discussion of the effects of Aβ_1–42_ on global cognition in the developed models will be presented in the next section.

### 3.4 Effects of CSF proteins on global cognition

#### 3.4.1 Alpha-synuclein

**Fig 4:**
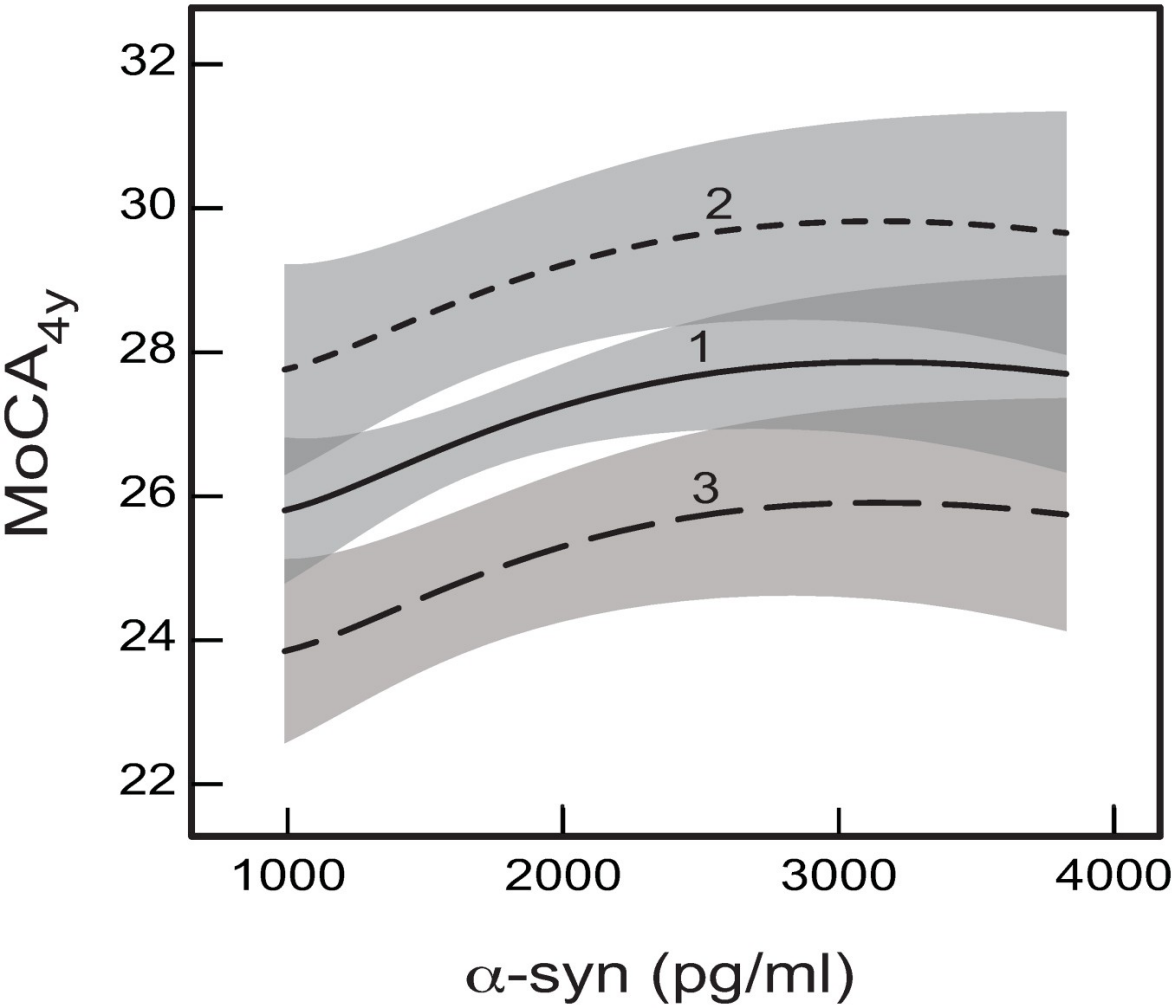
The predicted dependences of MoCA_4y_ on baseline CSF concentration of α-syn. The dependences result from the direct effects on MoCA_4y_ in the GSEM network given by Fig 1A for the following baseline ages: (1) Age = 60 years; (2) Age = 40 years; (3) Age = 80 years. The shaded bands indicate the 95% prediction intervals for the respective curves. The darker shade areas indicate overlaps of the prediction intervals for the neighboring curves. The other measures having direct effects on MoCA_4y_ were taken at their mean values: MoCA_b_ = 27, UPDRS_1-3_ = 31.87, GDS = 2.30, average ln(t-tau) = 3.71 (t-tau = 40.85 pg/ml), and average (Aβ_1–42_)^1/2^ = 18.95 (Aβ_1–42_ = 359.10 pg/ml).

For both the models (Figs 1A, 1B and 2), baseline CSF α-syn is predicted by the other CSF proteins, and it does not have any effects on any other considered baseline variables, but has an effect on MoCA_4y_. Therefore, the total effect of baseline α-syn on global cognition at 4 years after baseline is constituted by its direct (non-linear) effect on MoCA_4y_ (Fig 1A). As a result, there is no difference between the dependences of global cognition on baseline CSF concentration of α-syn for the two developed models, as the differences between the two models (Figs 1B and 2) do not influence the direct effect of α-syn on MoCA_4y_ (Fig 1A). Fig 4 shows the mutual dependences of MoCA_4y_ on baseline α-syn CSF concentration for both the developed models.

As can be seen within the shown range of α-syn between 1000 pg/ml and 4000 pg/ml, increasing baseline CSF concentration of α-syn results in better global cognition at 4 years after baseline for all considered baseline ages (Fig 4). This is consistent with the earlier findings to a similar effect [21, 24, 25, 32]. The curves for different ages (Fig 4) are parallel to each other, and increasing age expectedly results in decreasing MoCA_4y_ (Fig 4).

Note that the range of CSF α-syn in Fig 4 is considerably less than the overall range between ~ 330 pg/ml and ~ 6700 pg/ml for the considered sample of participants. This is because of the excessive statistical errors outside of the range shown in Fig 4. These errors are associated with the fact that the dependences in Fig 4 are plotted for the average values of the other proteins and, because of their interrelations, significantly increased or decreased values of α-syn are not likely to realistically correspond to the average values of the other proteins. As a result, the significant dependences could only be obtained around the central part of the α-syn range (Fig 4).

#### 3.4.2 Total tau

Similar to α-syn, the dependences of global cognition on CSF t-tau are the same for both the developed models (Figs 1A, 1B and 2). This is because the total effect of t-tau on MoCA_4y_ is the sum of the direct effect (Fig 1A) and one indirect effect through mediation α-syn (Figs 1B and 2). The differences between the two models (Figs 1A and 2) do not influence these effects.

**Fig 5:**
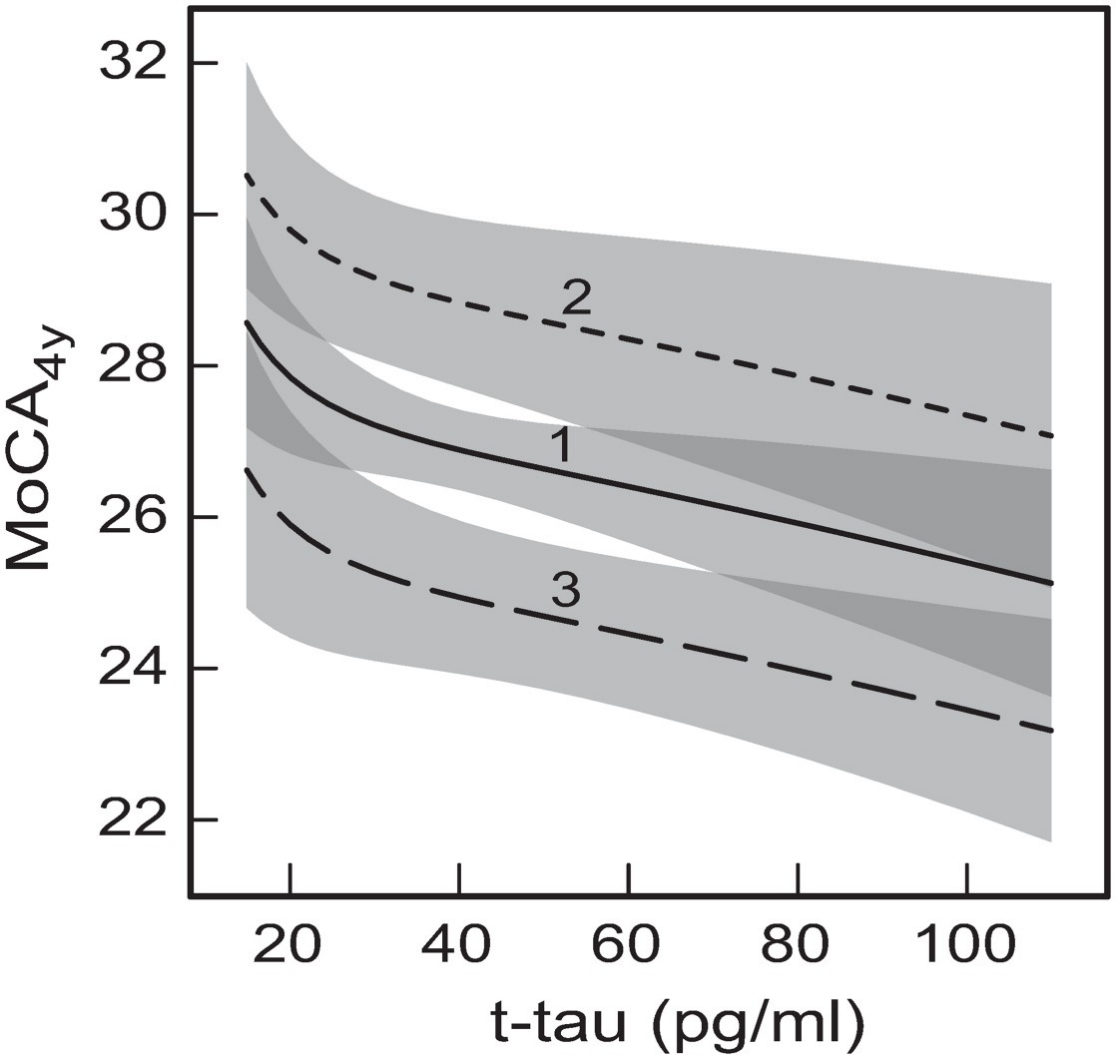
The predicted dependences of MoCA_4y_ on baseline CSF concentration of t-tau. The dependences result from the total effects of t-tau on MoCA_4y_ in either of the two GSEM networks for the following baseline ages: (1) Age = 60 years; (2) Age = 40 years; (3) Age = 80 years. The shaded bands indicate the 95% prediction intervals for the respective curves. The darker shade areas indicate overlaps of the prediction intervals for the neighboring curves. The other measures having direct effects on MoCA_4y_ were taken at their mean values: MoCA_b_ = 27, UPDRS_1-3_ = 31.87, GDS = 2.30, and average (Aβ_1–42_)^1/2^ = 18.95 (Aβ_1–42_ = 359.10 pg/ml), α-syn was predicted from (Aβ_1–42_)^1/2^ and ln(t-tau), as per the developed models.

At the same time, the cognition trends for CSF t-tau (Fig 5) are opposite to those for α-syn (Fig 4). Increasing baseline CSF concentration of t-tau results in monotonic decrease of MoCA_4y_ for all considered baseline ages (Fig 5). Some earlier studies also using the PPMI cohort of PD participants found that baseline CSF t-tau concentrations are lower in PD patients compared to healthy cohorts [25, 65]. This is not in contradiction with the trends of reducing level of cognition with increasing CSF t-tau concentration (Fig 5). It is quite possible that, on average, early PD patients (at baseline) do have lower levels of t-tau in CSF compared to healthy people, but higher levels of t-tau could, at the same time, cause more rapid cognitive decline (Fig 5). Furthermore, [25] also suggested that high CSF t-tau and low Aβ_1–42_ and α-syn might be a contributor to rapid progression of cognitive decline. This suggestion is consistent with the trend of worsening cognition under increasing CSF t-tau in early PD (Fig 5). The trends for baseline t-tau shown in Fig 5 are also consistent with the previously developed clinical scores for prediction of severe or mild-to-moderate cognitive decline in early PD [32], where increased baseline CSF t-tau resulted in larger probability of global cognitive decline in PD patients.

#### 3.4.3 Phosphorylated tau 181

**Fig 6:**
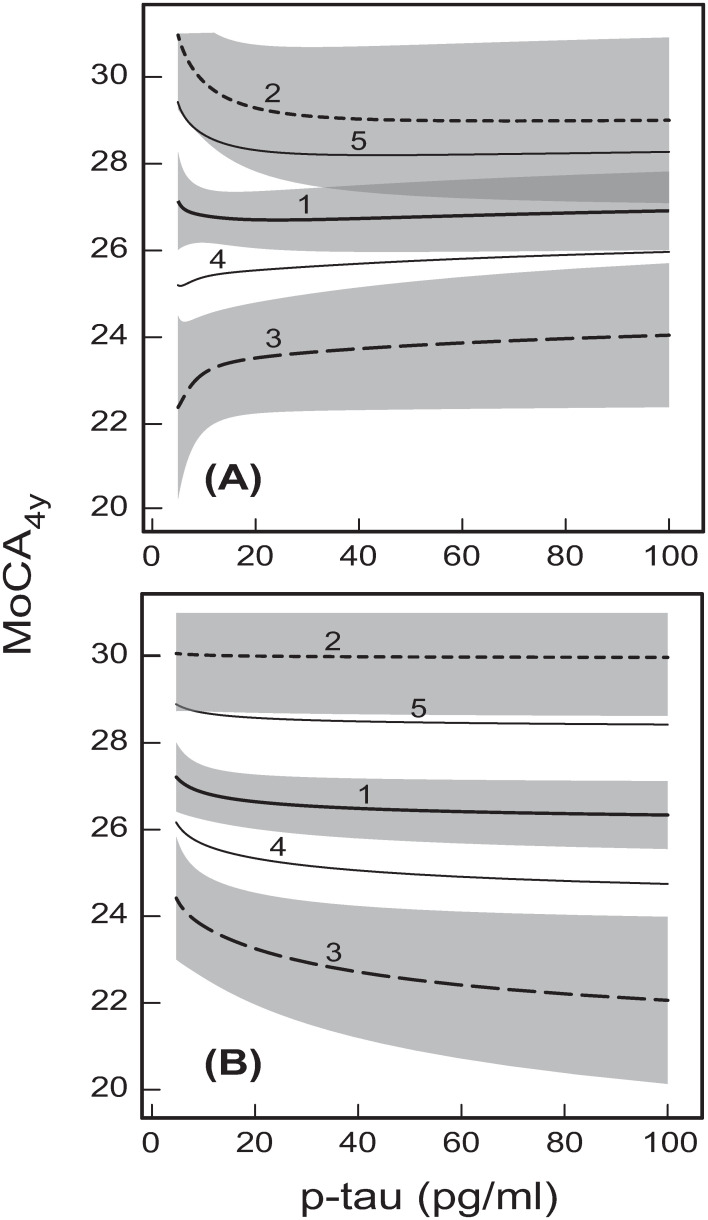
The predicted dependences of MoCA_4y_ on baseline CSF concentration of p-tau. The dependences result from the GSEM networks with: **(A**) CSF p-tau as the predicting protein variable (Fig 1A and 1B); and **(B**) CSF Aβ_1–42_ as the predicting protein variable (Figs 1A and 2). The curves are presented for the following baseline ages: (1) Age = 60 years; (2) Age = 40 years; (3) Age = 80 years; (4) Age = 70 years; and (5) Age = 50 years. The shaded bands indicate the 95% prediction intervals for the three curves 1, 2 and 3. For ease of visual perception, the prediction intervals are not shown for curves 4 and 5. The other variables for these graphs were either taken at their mean/base values: DaT = 1.38, Education = 15.64 years, Gender = Male, and GDS = 2.30 for (A) and (B), and also average (Aβ_1–42_)^1/2^ = 18.95 (Aβ_1–42_ = 359.10 pg/ml) for (B), or were predicted by the effect paths in the developed models (Figs 1A, 1B and 2).

The dependences of MoCA_4y_ on p-tau, corresponding to the total effects of p-tau on MoCA_4y_ in the two developed models (Figs 1A, 1B and 2), are shown in Fig 6. Expectedly, these dependences are significantly different for the two different models (compare Fig 6A and 6B). This is because p-tau does not have a direct effect on global cognition (Fig 1A). Therefore, the total effects of p-tau on MoCA_4y_ are the sums of all indirect effects for each of the networks in Figs 1B and 2. Because these networks are different, the relevant indirect effects are also different, which results in significant differences between Fig 6A and 6B.

The overall level of global cognitive function again expectedly decreases with increasing baseline age in both the models–compare curves 1–5 in Fig 6A and 6B. However, the trends for the global cognition scores at 4 years after baseline as a function of baseline CSF p-tau are different in the two models. For example, the baseline age of 60 years appears to be the threshold age separating the two opposite types of dependences of MoCA_4y_ on p-tau in the first model with p-tau as the triggering protein variable (curve 1 in Fig 6A). Below 60 years at baseline, increasing CSF p-tau results in declining global cognitive performance at 4 years after baseline (Fig 6A), whereas for Age > 60 years at baseline, global cognition at 4 years after baseline declines with decreasing p-tau (Fig 6A). At the baseline age of 60 years, there is hardly any dependence of MoCA_4y_ on baseline CSF p-tau concentration (Fig 6A). The diametrically opposite trends in the first model (Fig 1A) for older (> 60 years at baseline) and younger (< 60 years at baseline) PD patients could potentially explain the suggested inconsistencies of the earlier findings about CSF p-tau as a possible indicator of cognitive decline in PD [36, 58].

It could be hypothesized that at younger age the mechanisms of clearance of waste products, including phosphorylated tau, from the brain could be more effective [66], and this could halt cognitive decline caused by tau-mediated neurodegeneration even in the presence of diagnosable PD. It is possible to expect that at least some p-tau be cleared from the brain by the glymphatic flow [40, 67]. It is also possible to expect that the processes of immunological repair and clearance (by glial cells) of synapses and cell membranes damaged by Alzheimer’s pathology [40, 41, 64, 68, 69], and/or intracellular autophagy of neuronal tau tangles [40] could cause leakage of p-tau into the interstitial fluid, with its subsequent transport into, and clearance by, CSF [67]. Therefore, low levels of CSF p-tau in younger PD patients with adequate brain immune response could be indicative of its effective paravascular clearance, and this could be associated with better global cognitive function [67]–see curves 2 and 5 in Fig 6A. In addition, younger PD patients could also experience lower levels of hyperphosphorylation and tau-mediated neurodegeneration, and this could further contribute to the association of lower CSF levels of p-tau with better cognition (curves 2 and 5 in Fig 1A).

However, the adequate immune response in the brain is typically expected to diminish with age [40, 41]. Therefore, with increasing baseline age above 60 years (curves 3 and 4 in Fig 6A), low CSF p-tau levels could become more indicative of the lack of effective immunological repair and clearance of synaptic and neuronal damage, rather than of effective paravascular clearance that is also likely to decrease with age [66]. As a result, lower CSF levels of p-tau may no longer reflect more efficient drainage of toxic waste, but could rather become indicative of its more extensive deposition in the brain (due to age-impeded immunological clearance). This might lead to faster cognitive decline in PD patients with decreasing CSF p-tau in the model where p-tau is the predicting protein variable (curves 3 and 4 in Fig 6A).

The dependences of MoCA_4y_ on CSF concentration of p-tau in the second model (Fig 6B) show different trends to those in Fig 6A. In this case, the major trend for older patients is monotonic decline of the global cognitive function with increasing CSF p-tau concentration (opposite to Fig 6A). For younger patients, there is hardly any dependence of global cognition on p-tau (curves 2 and 5 in Fig 6B). The differences between the curves in Fig 6A and 6B could highlight the potentially different cognitive outcomes for the two hypothesized PD molecular pathways–with p-tau or Aβ_1–42_ as the alternative trigger CSF protein variables (Figs 1B and 2).

It follows from here that, when making predictions of cognitive decline in PD on the basis of CSF p-tau, it is essential to take account not only of patient’s age, but also of a possible molecular pathway, with either p-tau or Aβ_1–42_ acting as the trigger protein variable. It might, therefore, be difficult to use CSF p-tau as a biomarker for cognitive decline in PD until we are able to stratify the patients in accordance with the two molecular pathways (Figs 1B or 2), which is beyond the scope of the current paper.

As was highlighted above in Section 3.1, CSF p-tau has only indirect effects on cognition through mediation of other protein variables (Figs 1A, 1B and 2). As a result, whether or not p-tau is a primary triggering protein in the considered models (Figs 1A, 1B and 2), its total effects on global cognition are relatively small. It was also argued (Section 3.1) that this could be an explanation for the earlier propositions that the findings about phosphorylated tau as a biomarker of cognitive decline in PD were inconsistent [36, 58]. In addition to that, the observed heterogeneities of the dependences of MoCA_4y_ on p-tau for different ages and two different molecular pathways (Fig 6A and 6B) provide further explanation for the previously discussed [36, 58] inconsistencies of p-tau as a cognition biomarker in early PD.

#### 3.4.4 Amyloid beta

**Fig 7:**
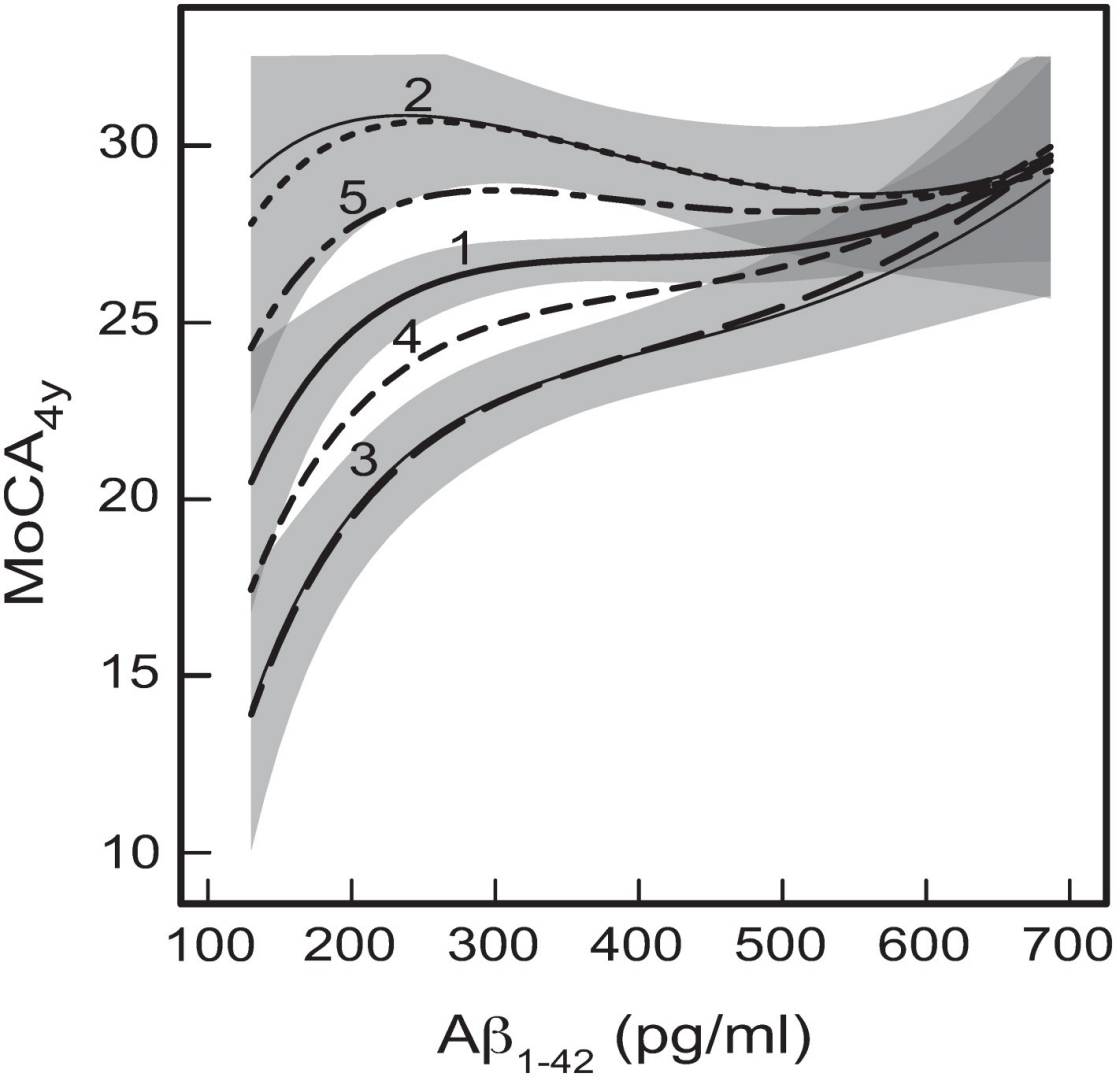
The predicted dependences of MoCA_4y_ on baseline CSF concentration of Aβ_1–42_. The dependences result from the GSEM networks given by Fig 1A and 1B (thick curves) and Figs 1A and 2 (thin solid curves) for the following baseline ages: (1) Age = 60 years; (2) Age = 40 years; (3) Age = 80 years; (4) Age = 70 years; and (5) Age = 50 years. The shaded bands show the 95% prediction intervals for the three thick curves 1, 2 and 3. The other variables for these graphs were either taken as their mean/base values: DaT = 1.38, Education = 15.64 years, Gender = Male, GDS = 2.30 for all the curves, and also average (p-tau)^-1/2^ = 0.283 (p-tau = 12.49 pg/ml) for thick curves 1–5, or were predicted by the effect paths in the developed models (Figs 1A, 1B and 2).

The typical dependences of MoCA_4y_ on baseline CSF concentration of Aβ_1–42_, resulting from the GSEM networks given by Figs 1A, 1B and 2, are shown in Fig 7 for five different baseline ages. It can be seen that the differences between the two models are rather minimal–compare the thin solid curves 2 and 3 in Fig 7 with the corresponding thick dotted and dashed curves 2 and 3. Further, for curve 1 in Fig 7 the differences between the predictions of the two models appeared to be less than ~ 2% (not shown in Fig 7). This demonstrates the close similarities between the two alternative models in terms of predicting global cognitive function in PD patients on the basis of CSF amyloid beta concentration. These predicting similarities can be explained by that the greatest contribution to MoCA_4y_ comes from the direct effects of Aβ_1–42_, α-syn, and t-tau (Fig 1A). The differences in the indirect effects of CSF proteins originating from the alternative models in Figs 1B and 2 introduce only small corrections (Fig 7).

Strong non-linear effects are obvious from the curves in Fig 7, which highlight the need for careful differentiation between different ranges of Aβ_1–42_. For example, it can be seen that for low values of Aβ_1–42_ (between ~ 130 pg/ml and ~ 200 pg/ml) the rates of changing MoCA_4y_ are notably higher than at > 250 pg/ml (Fig 7). Furthermore, for younger patients (with Age ≤ 60 years) and CSF Aβ_1–42_ concentrations > 250 pg/ml, the levels of global cognition at 4 years after baseline are virtually independent of amyloid beta concentration, or even reverse to negative slope (curve 2 in Fig 7). This is important for the adequate use of amyloid beta as a biomarker for clinical prediction of potential cognitive decline in PD patients. Not taking into account the demonstrated significant non-linearities and age differences for the dependences of MoCA on CSF amyloid beta could lead to failures of amyloid-based PD progression biomarkers.

Similar to Figs 4–6, we can also see that increasing baseline age generally results in a significant decrease of global cognitive function (Fig 7). However, this is not true for very high CSF concentrations of Aβ_1–42_, in which case there were no differences in cognition between different ages (Fig 7). It appears that particularly high levels of CSF amyloid beta concentration offer effective protection against not only PD-related but also age-related cognitive decline (Fig 7). This is very different from the previous dependences on p-tau, t-tau, or α-syn, where increasing baseline age resulted in significant reduction in global cognition for all values of those proteins (Figs 4–6). Further, whether the amyloid pathology is a trigger for tau and α-syn pathologies (Fig 2), or it is caused by the primary changes in p-tau (Fig 1B), does not change the final outcome of the crucial role of CSF amyloid beta in progression of cognitive decline in PD. In either model, the outcome is the same–high levels of CSF Aβ_1–42_ concentration are protective against cognitive decline, no matter the baseline age of patients (Fig 7).

These findings are consistent with the previous observations that Alzheimer’s comorbid pathology is associated with faster cognitive decline in PD patients [5, 35, 36], and with the amyloid hypothesis for Alzheimer’s pathology [40, 64]. We believe that the highlighted protective capability of high CSF amyloid beta concentrations demonstrates and confirms the unique role of amyloid in progression of cognitive decline in PD. It could be hypothesized that high levels of CSF amyloid beta concentration are associated with more effective degradation of insoluble amyloid beta depositions and plaques, resulting in more efficient removal of toxic proteins and peptides from the brain by way of the interstitial fluid and CSF [40, 67]. This could be protective against accumulation of toxic amyloid forms and insoluble depositions in the brain. A possible mechanism for this process could be the immunological activation of microglia and catabolism of amyloid beta [68–71].

The types of dependences of MoCA_4y_ on Aβ_1–42_ are different for baseline ages below and above 60 years (Fig 7). For older patients (> 60 years at baseline), decreasing CSF concentration of Aβ_1–42_ results in monotonic decline of global cognition at 4 years after baseline (curves 3 and 4 in Fig 7). For younger patients (< 60 years at baseline), the situation is different, with a maximum of global cognition being predicted at CSF Aβ_1–42_ concentrations of around 250–330 pg/ml (curves 2 and 5 in Fig 7), although these maxima in curves 2 and 5 are not statistically significant for the considered sample of participants. Nonetheless, the trend towards decreasing global cognition on the left of the maxima with decreasing CSF amyloid beta concentration (curves 2 and 5 in Fig 7) is consistent with the similar trend for older participants (curves 1, 3 and 4 in Fig 7). On the right of the cognition maxima (for larger CSF amyloid concentrations) global cognitive function remains approximately independent of Aβ_1–42_ for younger participants (curves 1, 2, and 5 in Fig 7).

As discussed above in this section, the observed tendency for older PD participants (with Age > 60 years) towards better cognition at 4 years after baseline with increasing baseline CSF Aβ_1–42_ concentration is also consistent with the previous similar findings relating the amyloids [20, 23, 25, 32, 36]. This trend becomes more obvious in the oldest patients (curves 3 in Fig 7), which could be due to the overall age-related decline in efficiency of the waste removal systems in the brain. The differences between the curves in Fig 7 highlight significant differences in the molecular processes in older and younger brains, as well as the potential existence of additional mechanisms/pathways for amyloid beta removal. In older patients, these additional mechanisms/pathways are more likely to fail, which is why lower CSF Aβ_1–42_ concentrations (i.e., potentially less efficient clearance by way of CSF) are more likely to result in accumulation of toxic amyloids and their insoluble formations (neuritic plaques) in the brain, contributing to cognitive decline (Fig 7). This hypothesis will require further confirmation in future studies focusing on molecular mechanisms of removal of amyloid beta from the brain, including any changes occurring in ageing brains.

It can also be seen that the characteristic ranges of changing MoCA_4y_ under variation of CSF Aβ_1–42_ concentration are significantly larger than those for α-syn (Fig 4), t-tau (Fig 5), and p-tau (Fig 6). Therefore, CSF Aβ_1–42_ concentration in the developed GSEM models (Figs 1A, 1B and 2) should be deemed as a better CSF protein biomarker for cognitive decline in PD, compared to α-syn, t-tau, and p-tau. This still does not mean that Aβ_1–42_ on its own is capable of accurately predicting cognitive decline in PD (possibly, apart from the extreme cases of its very high CSF concentrations, where cognitive decline does not occur–Fig 7). It could only be suggested that baseline Aβ_1–42_ is the best cognition marker out of the four considered CSF measures. Accurate prediction of cognitive decline should rather be conducted using the integrated biomarkers/scores involving weighed combinations of multiple individual biomarkers [32].

#### 3.4.5 Effects of other variables on global cognition

As can be seen from Fig 1A, there are multiple clinical and demographic variables, apart from Age and the four protein measures, which also influence the levels of global cognition of PD patients at baseline and 4 years later. The significant and large effect of MoCA_b_ on MoCA_4y_ is expected. It is quite logical that PD patients with better cognition at baseline will tend to retain (on average) better cognition at 4 years after baseline. This is because of a higher ‘starting point’ and/or potential protective effect of better baseline cognition and greater extent, complexity and redundancy of baseline neural networks.

The two demographic variables–Education and Gender–have only indirect effects on MoCA_4y_ through the mediation of MoCA_b_ (Fig 1A). There are no significant direct effects of these variables on MoCA_4y_. It could thus be concluded that the only role of Education and Gender is to contribute to the ‘starting point’ and/or baseline neural networks, and through this, they have indirect effects on the subsequent levels of global cognition 4 years later. Therefore, according to the developed models, these two variables can only modulate the initial conditions for PD progression.

**Fig 8:**
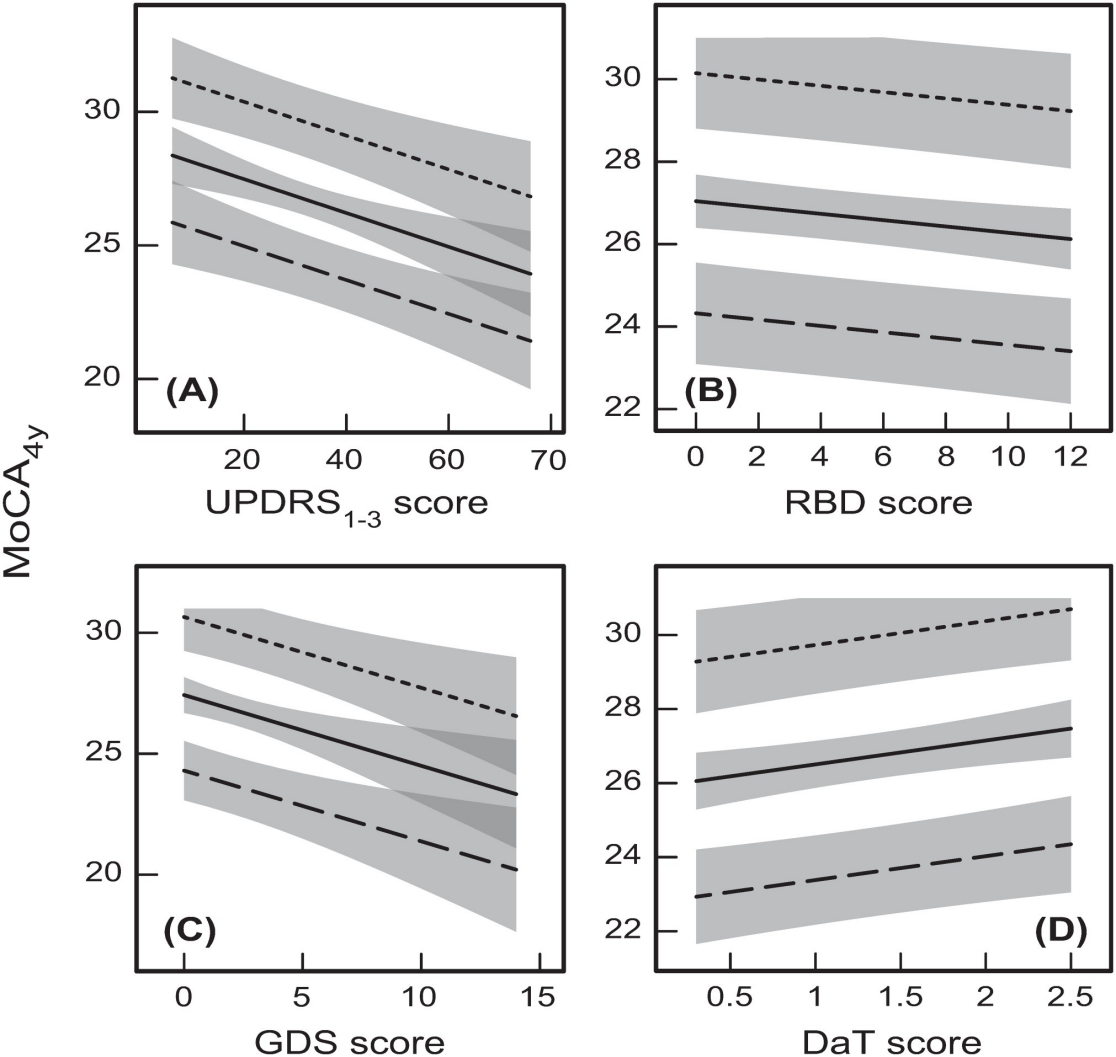
The predicted dependences of MoCA_4y_ on the four baseline clinical measures/scores. **(A)** UPDRS_1-3_; **(B)** RBD; **(C)** GDS; **(D)** DaT for the three values of Age = 40 years (dotted lines), 60 years (solid lines), and 80 years (dashed lines). The other variables for these graphs were either taken as their mean/base values: Education = 15.64 years, Gender = Male, average (Aβ_1–42_)^1/2^ = 18.95 (Aβ_1–42_ = 359.10 pg/ml), average ln(α-syn) = 7.43 (α-syn = 1685.81 pg/ml), average ln(t-tau) = 3.71 (t-tau = 40.85 pg/ml), and **(A)** GDS = 2.30; **(B)** GDS = 2.30, DaT = 1.38; **(C)** DaT = 1.38; and **(D)** GDS = 2.30, or predicted by the respective effect paths in the developed model (Fig 1A).

The typical dependences of MoCA_4y_ on the other 4 clinical variables/measures (due to their total effects on MoCA_4y_) are shown in Fig 8. In particular, it can be seen that the greatest (out of these four variables) effects on MoCA_4y_ come from UPDRS_1-3_ and GDS, and these effects are around 5 points on the MoCA scale over the whole ranges of UPDRS_1-3_ and GDS (Fig 8A and 8C). The effects of the other two variables (RBD and DaT) are about 5 times smaller (Fig 8B and 8D). This is because neither RBD, nor DaT have significant direct effects on MoCA, and this significantly reduces their contribution to changing MoCA_4y_.

It might not be considered as surprising that UPDRS_1-3_ has a large significant effect on MoCA_4y_, because UPDRS_1-3_ is a PD-specific measure closely related to the disease progression. However, the approximately equally large contribution from GDS could be regarded as more unexpected, because this variable is not necessarily PD-specific. This is a demonstration of a close and strong relationship between depression and progression of cognitive decline in PD, which is supported by the strong direct effect of GDS on MoCA_4y_ (Fig 1A). According to our findings, geriatric depression tends to significantly accelerate cognitive decline after PD diagnosis, which constitutes PD progression.

Because MoCA_b_ is a baseline measure, it is close to characterizing the global cognitive function prior to PD. The lack of a direct effect of GDS on MoCA_b_ (Fig 1A) could thus be seen as an indication that geriatric depression is not as important for cognition in the absence of PD or at its prodromal stages. The only indirect effects of GDS on MoCA_b_ are relatively weak and come through mediation of UPDRS_1-3_ and RBD (Fig 1A). The presence of these indirect effects is a further confirmation of the impact of GDS on PD progression, but not on cognition prior to acquiring PD. Indeed, GDS has direct effects on UPDRS_1-3_ and RBD, which then have effects on MoCA_b_ (Fig 1A). Because the UPDRS_1-3_ and RBD scores are rather PD-specific variables, their abnormalities suggest likely PD progression (even if the disease is at its early stage). Therefore, the indirect effects of GDS on MoCA_b_ through mediation of UPDRS_1-3_ and RBD (Fig 1A) could also be regarded as consequences of PD progression.

It could thus be suggested that the effect of geriatric depression on cognition is strongly enhanced by PD. While the effect of depression on cognition in the absence of PD is small (if any), the presence of PD drastically changes the situation, and GDS becomes a major contributor to cognitive decline (Fig 8C). A possibility of such enhancement was proposed earlier, although not proven, possibly due to the limitations of the considered small sample and the adopted analytical approach based on group comparisons [72].

Confounding effects of coexisting or comorbid psychological stress (e.g., caused by PD burden or other factors) are hypothesized to be one of the possible explanations of the strong effects of GDS on cognitive decline during PD progression. Yang, et al. [73] highlighted that a large part of depressed patients display characteristic endocrine and immune markers of psychological stress (including elevated levels of cortisol and pro-inflammatory cytokines). Further, Burke, et al. [74] found that even if in the absence of stress depressed and non-depressed people display similar levels of plasma cortisol, depressed patients have much higher cortisol levels during the stress recovery period. On the other hand, the effects of psychological stress on bodily functions could be enhanced by the presence of an underlining condition also affecting these functions–similarly to the enhancement of the effect of severe psychological stress on blood parameters in the presence of the serious blood disorder [75]. Therefore, whether psychological stress is associated with depression or caused by additional confounding effects not considered in this study (e.g., by PD burden), its effects on cognition could be significantly enhanced by the presence of depression (higher GDS score) and the underlining illness–PD. Further targeted research will be needed to confirm or otherwise this stress-related hypothesis for the influence of depression on cognition in PD. The analysis of possible effects of GDS on other motor and non-motor symptoms in PD will also be of an interest, but is beyond the scope of the current paper.

Irrespectively of the proposed stress hypothesis as a possible mechanism for the impacts of geriatric depression on the rate of cognitive decline in early PD, the obtained findings suggest geriatric depression as one of the major factors accelerating cognitive decline in early PD patients. A significant clinical outcome from this finding is the potential need for effective treatment of geriatric depression in early PD, which could counter or slow dawn PD progression in the global cognitive domain.

#### 3.4.6 Conclusions

In this paper, we used the PPMI database of PD patients at early stages of the disease to develop and justify networks of effects between 14 baseline demographic, clinical, and pathological measures to understand their direct and indirect effects (effect paths) on global cognition in PD patients at baseline and 4 years later. The complex patterns of the indirect effects revealed in this study demonstrated the intricate interrelations between the considered variables. Significant non-linearities of some of these effects must be taken into account when predicting global cognition and its decline in PD patients.

Total effects of the considered variables on global cognition at baseline and 4 years later were calculated and interpreted. These outcomes allowed deeper insights into the roles of the considered variables in the process of decline of the global cognitive function in PD. In particular, it was demonstrated that the total (negative) effect of GDS on global cognition 4 years after baseline was approximately the same as that of UPDRS_1-3_, i.e., around 5 points on the MoCA scale over the full ranges of the GDS or UPDRS_1-3_ scores. This highlighted the important role of depression in PD progression, but not at the time of diagnosis or during the prodromal stages of PD.

The developed GSEM networks allowed identification and characterization of potential molecular triggers and pathways for PD progression at early stages of the disease. One of the developed networks suggested CSF p-tau as the trigger protein variable predicting the behaviour of the other three protein variables and cognitive decline in PD. The other model suggested CSF Aβ_1–42_ as the triggering variable predicting p-tau, t-tau, and α-syn. It was hypothesized that the two alternative models might either correspond to two different PD sub-types, or reflect the two different types of processes occurring concurrently in PD patients or some of the patients from the considered cohort. Further research will be needed to clarify this issue.

Neither of the two alternative models indicated CSF α-syn as the primary protein trigger for PD, which means that synucleinopathy could be secondary to the triggering primary changes involving amyloid beta and misfolded tau proteins. According to the developed models, it is possible that, once the pathological process has been initiated by the involvement of p-tau and/or Aβ_1–42_, cross-fibrillization of tau, α-synuclein and amyloids could occur [35, 61, 62]. This is consistent with the prion theory of neurodegenerative diseases [38, 39, 57] and synergetic nature of α-syn neuropathology and Alzheimer’s disease neuropathology in PD [35, 36, 61, 62].

Further, it was shown that baseline CSF Aβ_1–42_ is likely to be the best individual protein marker for prediction of cognitive decline in PD, with high levels of CSF Aβ_1–42_ offering major protection against cognitive decline, no matter the baseline age of the PD patients. Baseline CSF concentrations of Aβ_1–42_ exceeding 500 pg/ml were associated with nearly full protection against cognitive decline at 4 years after baseline for all considered baseline ages between 40 and 80 years. Whether CSF amyloid beta is a trigger for tau and α-syn pathologies, or it is caused by the primary changes in CSF p-tau, does not matter for the final outcome of the crucial role of amyloids in progression of cognitive decline in early PD. It could thus be proposed that future development of therapeutic interventions for early PD could be aimed at increasing CSF concentration of amyloid beta as a mechanism for its effective removal from the brain, with the resulting possible protection against cognitive decline. In particular, it would be interesting to see if effective microglial activation, e.g., by triggering receptor expressed on myeloid cells 2 (TREM2) [68–71], or antibody therapies targeting amyloid-beta plaques could be options to achieve this. In addition, further research will be needed to establish the exact molecular/immunological mechanisms responsible for high CSF levels of Aβ_1–42_ in some of early PD patients.

The clinical value of the current study arises from the gained new knowledge about the fundamental relationships between multiple individual markers of cognitive decline in early PD. This knowledge will enable more effective development of meaningful biomarkers and methods for more reliable prediction of risks of cognitive decline in individual PD patients. It will assist with the identification and characterisation of the most important individual factors (such as, for example, CSF levels of amyloid beta and geriatric depression score) that present the highest risks for progression of cognitive decline in PD. Such knowledge will inform the development of more effective therapeutic approaches halting or even reversing PD progression. For example, based on the obtained outcomes, therapies increasing CSF levels of amyloid and effectively treating depression at early stages of PD could assist with suppressing PD-related cognitive decline. The findings of this study will also be important for optimal design of clinical trials through well-based stratification of the participants and their subsequent evaluation.

The main limitations of the study include the reliance on the single PPMI cohort of 269 participants. Future multi-cohort validation of the developed effect networks and total effects of the considered variables on global cognition in PD will be beneficial. The study was limited to the 14 variables and measures available from the PPMI database. EGF, cholesterols and triglycerides were not involved in this study due to the respective sample size limitations. The four protein PD markers were evaluated in CSF. Although it is expected that the obtained relationships between these CSF markers are reflective of the processes in the brain, direct extensions of these relationships and causalities to the processes in the brain should be done with caution, as changes in the brain might not be equivalent at all times to changes in CSF. The latest findings highlighted the superior performance of other types of tau phosphorylated at threonine-217 and threonine-231 as potential biomarkers for Alzheimer’s disease [76, 77]. Although it will be interesting to consider these types of phosphorylated tau in the future models, this was not done in the current study due to the lack of the respective data in the available cohort. The evaluation of the global cognitive function was based on the MoCA scale. This could be another potential limitation, although the MoCA scale is widely recognized as a valid instrument for evaluation of the global cognitive function including in PD patients [25, 30, 31, 47, 48]. As discussed above in the body of the paper, the possibility of learning how to answer the MoCA questions (during multiple repeated use of the MoCA scale) by participants with better cognition and learning abilities could be a source of potential bias for the outcomes at 4 years after baseline. As the obtained findings are relevant to global cognition and its possible decline in early PD, their extension to specific cognitive domains might need further studies and justification. The determination and analysis of networks of effects for potential biomarkers with regard to progression of non-cognitive PD symptoms was beyond the scope of the current study. Finally, the PPMI baseline data was collected within 2 years after the initial PD diagnosis. Therefore, the developed models (Figs 1B and 2) are limited to the consideration of the baseline parameters and any interrelations between them within this particular timeframe after the diagnosis. Consideration of earlier or later networks of effects in prodromal or more advanced PD will require further investigation in the future.

## Supporting information

S1 Text Supporting information for the GSEM models. S1 Text involves three sections: (Section 1) Figure demonstrating the censored nature of the distribution of the MoCA data at baseline and four years later; (Section 2) Development and justification of the two alternative GSEM models; and (Section 3) Lists of significant indirect effects in the developed GSEM models; (Section 4) Validations of the GSEM models; (Section 5) Supporting References in S1 Text.
(DOCX)
