# Supplementary material for: Path analysis of biomarkers for cognitive decline in early Parkinson’s disease
Source: PLoS One. 2022 May 13;17(5):e0268379. doi: 10.1371/journal.pone.0268379 (PMC9106174; doi:10.1371/journal.pone.0268379)
Supplement: S1 Text — involves three sections: (Section 1) Figure demonstrating the censored nature of the distribution of the MoCA data at baseline and four years later; (Section 2) Development and justification of the two alternative GSEM models; and (Section 3) Lists of significant indirect effects in the developed GSEM models; (Section 4) Validations of the GSEM models; (Section 5) Supporting References in S1 Text. (DOCX) [file pone.0268379.s001.docx]

S1 Text: Supporting Information for the GSEM models

# Supporting Figure

Demonstration of the censored (from the right) nature of the distribution of the MoCA data at baseline and 4 years later.


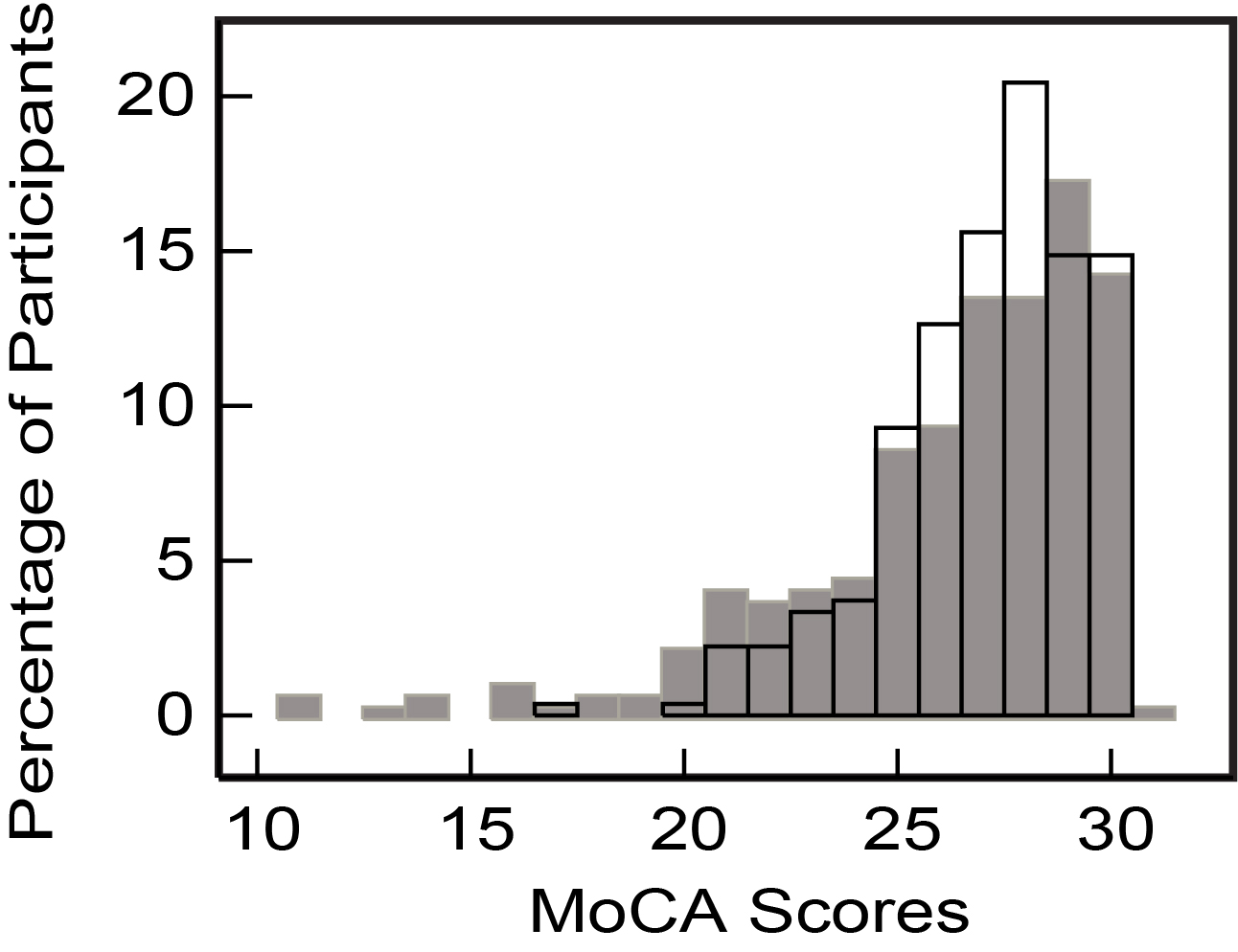


**Figure.** Percentage distributions of the MoCA scores at baseline (empty columns) and 4 years later (grey columns). The width of the bins for the MoCA scores was 2 points on the MoCA scale.

# Development of GSEM Models

Several aspects were taken into account when determining the directions (possible causality) of the direct effects in the GSEM model. Firstly, it was unreasonable to expect that any of the considered clinical or pathological measures were able to influence such variables as baseline age (Age), years of prior education, and gender. Therefore, it was assumed that the direction of any significant effects should be from these three variables towards the MoCA scores, clinical and pathological measures. The pathological and DaT measures were considered as the physiological measures of PD and/or geriatric pathology. Variations of these measures could be regarded as causing changes in the observed symptomatic manifestations measured by UPDRS_1-3_, RBD, and GDS. Therefore, it was assumed that any significant effects should be directed from the pathological and DaT measures towards the clinical measures, such as UPDRS_1-3_, RBD, and GDS. It was also possible to expect that the effect direction should be considered from GDS to other clinical measures (such as RBD and UPDRS_1-3_). This is because GDS is the general measure for geriatric depression, rather than a specific measure for PD. It was useful and logical to consider effects from GDS on RBD and UPDRS_1-3_ that are more PD-specific. In addition, for example, using the reversed effect from RBD on GDS increases the Akaike’s information criterion (AIC) [s1] of the model by 16.6%, which demonstrates significantly worse model fit.

Secondly, the directions of any significant effects between the considered variables were also governed by the following procedure of GSEM development. The first step was to use the multiple regression approach to identify any demographic, clinical and pathological measures having significant direct effects on the MoCA_b_ and MoCA_4y_ scores. For example, using the forward 0.05/4

tion and backward elimination approach, it was found that UPDRS_1-3_ had a significant direct effect on MoCA_b_ and MoCA_4y_, whereas RBD and DaT did not. Therefore, the second step was to identify any demographic, clinical and pathological measures having significant direct effects on UPDRS_1-3_. In particular, it was found that both RBD and DaT had significant effects on UPDRS_1-3_, which determined the directions of these effects from RBD and DaT towards UPDRS_1-3_. The directions of other direct effects between the considered variables were determined in a similar fashion, taking also into account the arguments presented in the previous paragraph.

However, the arguments discussed in the two paragraphs above were still not enough for the determination of a justifiable network for the four transformed CSF measures: ln(t-tau), ln(α-syn), (Aβ_1-42_)^1/2^, and (p-tau)^-1/2^. This is because of the possible significant synergy between these four measures [21,35-37] and lack of solid information as to which of them could be regarded as producing causal impacts on the others. Therefore, the adopted analytical methodology for the derivation of a justifiable network of significant effects involving the four CSF measures was based on modification indices for structural equation modelling (SEM) in Stata16 [34]. As demonstrated below, these modification indices allow justified mathematical choice of improvements to SEM model step-by-step from a very basic model to the one with a developed sophisticated network of potentially causal effects between the involved variables.

Note that we used SEM for this derivation, because GSEM that was used for the final models (Figs. 1 and 2) does not allow the use of modification indices [34]. However, in SEM, only linear effects could be considered, with no non-linear terms or pair-wise interactions between the variables [34] (some of which were significant in the final models – Figs. 1 and 2). Therefore, the derivation of the final networks of effects (involving non-linearities and interactions) between the four CSF measures (Figs. 1B and 2) was undertaken in two major steps. Firstly, valid networks of linear effects involving the four CSF measures and baseline age were developed using SEM with modification indices (see below). This allowed the determination of the likely directions of the effects between these four CSF measures and Age. Subsequently, the developed SEM networks were checked, using the GSEM approach, for the involvement of any significant non-linearities and interactions between the variables, and the final networks shown in Figs. 1B and 2 were developed.

This network derivation started from the simple regressions determining the effects of baseline age on each of the four CSF measures separately. As a result, only the Age effect on ln(t-tau) was significant, with *p* < 0.001, whereas the effects of Age on the other three CSF measures ln(α-syn), (Aβ_1-42_)^1/2^, and (p-tau)^-1/2^ were not significant, with the respective *p*-values of 0.099, 0.059, and 0.416. The levels of significance (under 10%) of the effects of Age on ln(α-syn) and (Aβ_1-42_)^1/2^ were indications that baseline age could have real effects on these variables (which was consistent with the earlier studies [35,s2,s3].

Nonetheless, because of the highest significance of the effect of baseline age on ln(t-tau), we commenced the derivation from the simplest SEM model with only one effect of Age on ln(t-tau), whereas all other CSF measures were considered as constants. Certainly, this model was not appropriate for an adequate description of the four CSF measures and baseline age. One of its most important goodness of fit (GOF) indices χ^2^ = 315, with *p* ≤ 0.0001, and this was a strong indication of inadequacy of the considered model to fit the available data. Therefore, it was only considered as a starting point (model M_1_) in our derivation of much better final SEM models. To proceed with this derivation, we calculated the modification indices (MIs) [34] for model M_1_. MIs suggest by how much the value of χ^2^ could be reduced upon involvement of other possible effects between the considered variables (smaller values of χ^2^ and larger *p*-values correspond to a better model) [34].

As an example, Table 1 in S1 Text shows the modification indices for model M_1_ involving only one effect of Age on ln(t-tau). It could be seen that, involving an additional effect of ln(t-tau) on ln(α-syn) in the model was to result in decreasing the value of χ^2^ by 110.6 (Table 1 in S1 Text). This is the maximum possible decrease in χ^2^ (maximum possible improvement of M_1_) upon involvement of one additional effect. The involvement of the inverse effect ln(α-syn) on ln(t-tau), or the covariance between these variables, would result in somewhat smaller decrease in χ^2^ (by 109.0 –Table 1 in S1 Text). Although the difference between these modification indices is rather small, this is still an indication of the preferred direction (potential/partial causality) of the effect from ln(t-tau) on ln(α-syn), because the reverse effect or the covariance between these variables result in a less pronounced improvement of M_1_. Accordingly, one effect from ln(t-tau) on ln(α-syn) was added to the initial model. The resultant model M_2_ contained the two effects: Age 🡪 ln(t-tau) and ln(t-tau) 🡪 ln(α-syn).

**Table 1 in S1 Text. Modification indices for initial model M_1_ with only one effect:**

**Age 🡪 ln(t-tau).**

| **Proposed Dependent Variable** | **Proposed Independent Variable** | **Modification Index (MI)** |
| --- | --- | --- |
| ln(α-syn) | ln(t-tau) | 110.6 |
|  | (Aβ_1-42_)^1/2^ | 34.1 |
|  | (p-tau)^-1/2^ | 33.2 |
| ln(t-tau) | ln(α-syn) | 109.0 |
|  | (Aβ_1-42_)^1/2^ | 45.9 |
|  | (p-tau)^-1/2^ | 73.6 |
| (Aβ_1-42_)^1/2^ | ln(α-syn) | 34.1 |
|  | ln(t-tau) | 36.0 |
|  | (p-tau)^-1/2^ | 46.3 |
| (p-tau)^-1/2^ | ln(α-syn) | 33.2 |
|  | ln(t-tau) | 71.8 |
|  | (Aβ_1-42_)^1/2^ | 46.3 |
| **Proposed Covariances** | | |
| ln(α-syn) & ln(t-tau) | | 109.0 |
| ln(α-syn) & (Aβ_1-42_)^1/2^ | | 34.1 |
| ln(α-syn) & (p-tau)^-1/2^ | | 33.2 |
| ln(t-tau) & (Aβ_1-42_)^1/2^ | | 45.9 |
| ln(t-tau) & (p-tau)^-1/2^ | | 73.6 |
| (Aβ_1-42_)^1/2^ & (p-tau)^-1/2^ | | 46.3 |

MIs were recalculated for model M_2_, and showed that the maximum improvement of the model could be achieved through the inclusion of one additional effect from (p-tau)^-1/2^ to ln(t-tau), for which MI = 73.6. The reverse effect from ln(t-tau) to (p-tau)^-1/2^ was to result in a smaller improvement of the model (by MI = 71.8). This was an indication of the preferred direction (possible/partial causality) of the effect (p-tau)^-1/2^ 🡪 ln(t-tau). Therefore, this additional effect was added to M_2_, thus resulting in a new model M_3_ with three effects: Age 🡪 ln(t-tau), (p-tau)^-1/2^ 🡪 ln(t-tau), and ln(t-tau) 🡪 ln(α-syn).

MIs were recalculated for M_3_. They showed that the maximum improvement of this model could be achieved by adding either of the two opposite effects: (p-tau)^-1/2^ 🡪 (Aβ_1-42_)^1/2^, or (Aβ_1-42_)^1/2^ 🡪 (p-tau)^-1/2^. For both these opposite effects MIs were the same and equal to 46.3 (the same decrease in χ^2^ value by 46.3 upon inclusion of either of these effects in M_3_). This did not allow the determination of a preferred direction of the effect between these two variables. Therefore, both paths for the development of the final model were used to develop two different final models. As a result, there were two different versions of M_4_: M_4_′ with the effect (p-tau)^-1/2^ 🡪 (Aβ_1-42_)^1/2^, and M_4_′′ with the effect (Aβ_1-42_)^1/2^ 🡪 (p-tau)^-1/2^.

MIs were recalculated for both models M_4_′ and M_4_′′, and models M_5_′ and M_5_′′ were obtained by including the additional effect (Aβ_1-42_)^1/2^ 🡪 ln(t-tau) with the largest MIs (of 16.1) corresponding to the largest improvement of either of the models M_4_′ and M_4_′′. This additional effect appeared to be the same for both the models M_5_′ and M_5_′′, although this should not have been necessarily expected in advance.

Similarly, models M_6_′ and M_6_′′ were obtained by adding the additional effect (Aβ_1-42_)^1/2^ 🡪 ln(α-syn) with MI = 7.59 to each of the models M_5_′ and M_5_′′. After this step, M_6_′′ could not be further improved, as there were no further MIs for that model. Therefore, M_6_′′ was the final SEM model with the following direct effects: Age 🡪 ln(t-tau); (p-tau)^-1/2^ 🡪 ln(t-tau); ln(t-tau) 🡪 ln(α-syn); (Aβ_1-42_)^1/2^ 🡪 (p-tau)^-1/2^; (Aβ_1-42_)^1/2^ 🡪 ln(t-tau); and (Aβ_1-42_)^1/2^ 🡪 ln(α-syn). This model resulted from the choice of the effect (Aβ_1-42_)^1/2^ 🡪 (p-tau)^-1/2^ at the step of the construction of model M_4_′′ - see above.

On the contrary, M_6_′ was not the final version of the model with the effect (p-tau)^-1/2^ 🡪 (Aβ_1-42_)^1/2^ at the step of the construction of model M_4_′. The recalculated MIs for M_6_′ suggested one additional improvement of the model by way of inclusion of the effect of Age on (Aβ_1-42_)^1/2^. This was consistent with the previous finding from the simple regression between (Aβ_1-42_)^1/2^ and Age, which resulted in *p* = 0.059 – see above. Therefore, if the effect (p-tau)^-1/2^ 🡪 (Aβ_1-42_)^1/2^ was chosen at the stage of construction of M_4_′, the final SEM model for the four CSF measures and baseline age was M_7_′ with the following direct effects: Age 🡪 ln(t-tau); Age 🡪 (Aβ_1-42_)^1/2^; (p-tau)^-1/2^ 🡪 ln(t-tau); ln(t-tau) 🡪 ln(α-syn); (p-tau)^-1/2^ 🡪 (Aβ_1-42_)^1/2^; (Aβ_1-42_)^1/2^ 🡪 ln(t-tau); and (Aβ_1-42_)^1/2^ 🡪 ln(α-syn). No more MIs were available for this model in the STATA16 software.

Thus, we obtained two different SEM models M_7_′ and M_6_′′ with the two different networks of effect for the four CSF measures and Age. In M_7_′, the primary protein variable was p-tau, with no effects on it from the other variables, whereas in M_6_′′, the primary protein variable was Aβ_1-42_, with no effects on it from the other variables. In other words, for M_7_′, the potential driving force for the pathogenic processes in PD was p-tau, whereas for M_6_′′, such potential driving force appeared to be Aβ_1-42_.

In an attempt to choose between these two final models, we considered the Akaike’s information criterion (AIC) [s1] and goodness of fit indices that characterize the suitability of an SEM model for the description of the available data. AIC was smaller for M_7_′ (AIC = 2652.7) than for M_6_′′ (AIC = 2653.9), which meant that model M_7_′ was somewhat better than M_6_′′. However, the difference was quite small and did not allow definitive conclusions as to which of the two models was actually better. Further comparison was conducted on the basis of the goodness of fit (GOF) indices for the two models (Table 2 in S1 Text). According to all 6 major GOF indices, the final model M_7_′ was better for the description of the available data. In particular, the value 0.427 of the coefficient of determination for M_7_′ meant that the developed SEM model could explain around 42.7% of the total model variance (which was an encouraging outcome, particularly taking into account that no any potential non-linearities or interactions have yet been involved in the model). This suggested that p-tau was more likely to be the primary protein variable (potential driving force for the pathogenic processes in PD) for the considered cohort of participants, compared to the other three CSF measures including Aβ_1-42_.

**Table 2 in S1 Text. Model fits for the final alternative SEM models.**

| **GOF indices** | **ALTERNATIVE FINAL SEM MODELS** | |
| --- | --- | --- |
|  | **M_7_′** | **M_6_′′** |
| *p*-value for χ^2^ | 0.524 | 0.209 |
| RMSEA | < 0.001 | 0.045 |
| CFI | 1 | 0.995 |
| TLI | 1 | 0.986 |
| SRMR | 0.010 | 0.041 |
| CD | 0.427 | 0.324 |

M_7_′ with the effect (p-tau)^-1/2^ 🡪 (Aβ_1-42_)^1/2^ (leading to the network in Fig. 1B); and M_6_′′ with the effect (Aβ_1-42_)^1/2^ 🡪 (p-tau)^-1/2^ (leading to the network in Fig. 2).

At the same time, although it was possible to see that model M_7_′ was better than M_6_′′ for the description of the available data, both the models had acceptable model fit demonstrating their suitability for the data. Therefore, neither of the two models could be excluded from the consideration on the basis of poor model fit, and both of them were considered and interpreted in this paper.

The presented statistical derivation of the final SEM models, based on the use of MIs, enabled us to justify the directions of the direct effects in the two alternative networks for the four CSF measures and baseline age. However, the SEM approach did not allow involvement of non-linear effects and/or any possible interactions between the involved variables [34]. At the same time, it was quite possible to expect that at least some of the considered direct effects between the CSF measures and baseline age could be significantly non-linear, and there could also be significant interactions between these variables. Therefore, the developed networks M_7_′ and M_6_′′ were additionally checked using the GSEM approach [34] for any possible non-linearities of the direct effects and possible pair-wise interactions between the variables. As a result, SEM model M_7_′ was transformed into the GSEM network shown in Fig. 1B, and SEM model M_6_′′ was transformed into the GSEM network shown in Fig. 2. Both of these GSEM networks contained significant non-linear effects and significant interaction terms (Figs. 1B and 2). The effects of ln(t-tau), (Aβ_1-42_)^1/2^, and ln(α-syn) on MoCA_4y_ are shown in Fig. 1A.

# Significant Indirect Effects in the GSEM Models

The significant (*p* < 0.1) indirect effects on MoCA_4y_ resulting from the first developed GSEM model (Figs. 1A,B) can be listed as follows:

Age 🡪 MoCA_b_ 🡪 MoCA_4y_;

Age 🡪 ln(t-tau) 🡪 MoCA_4y_;

Age 🡪 ln(t-tau) 🡪 ln(α-syn) 🡪 MoCA_4y_;

Age 🡪 (Aβ_1-42_)^1/2^ 🡪 MoCA_4y_;

Age 🡪 (Aβ_1-42_)^1/2^ 🡪 ln(t-tau) 🡪 MoCA_4y_;

Age 🡪 (Aβ_1-42_)^1/2^ 🡪 ln(t-tau) 🡪 ln(α-syn) 🡪 MoCA_4y_;

Age 🡪 (Aβ_1-42_)^1/2^ 🡪 ln(α-syn) 🡪 MoCA_4y_;

Age 🡪 (Aβ_1-42_)^1/2^ 🡪 RBD 🡪 UPDRS_1-3_ 🡪 MoCA_4y_;

Age 🡪 (Aβ_1-42_)^1/2^ 🡪 RBD 🡪 UPDRS_1-3_ 🡪 MoCA_b_ 🡪 MoCA_4y_;

Age 🡪 UPDRS_1-3_ 🡪 MoCA_4y_;

Age 🡪 UPDRS_1-3_ 🡪 MoCA_b_ 🡪 MoCA_4y_;

(p-tau)^-1/2^ 🡪 (Aβ_1-42_)^1/2^ 🡪 MoCA_4y_;

(p-tau)^-1/2^ 🡪 (Aβ_1-42_)^1/2^ 🡪 ln(t-tau) 🡪 MoCA_4y_;

(p-tau)^-1/2^ 🡪 (Aβ_1-42_)^1/2^ 🡪 ln(t-tau) 🡪 ln(α-syn) 🡪 MoCA_4y_;

(p-tau)^-1/2^ 🡪 (Aβ_1-42_)^1/2^ 🡪 ln(α-syn) 🡪 MoCA_4y_;

(p-tau)^-1/2^ 🡪 (Aβ_1-42_)^1/2^ 🡪 RBD 🡪 UPDRS_1-3_ 🡪 MoCA_4y_;

(p-tau)^-1/2^ 🡪 (Aβ_1-42_)^1/2^ 🡪 RBD 🡪 UPDRS_1-3_ 🡪 MoCA_b_ 🡪 MoCA_4y_;

(p-tau)^-1/2^ 🡪 ln(t-tau) 🡪 MoCA_4y_;

(p-tau)^-1/2^ 🡪 ln(t-tau) 🡪 ln(α-syn) 🡪 MoCA_4y_;

ln(t-tau) 🡪 ln(α-syn) 🡪 MoCA_4y_;

(Aβ_1-42_)^1/2^ 🡪 ln(t-tau) 🡪 MoCA_4y_;

(Aβ_1-42_)^1/2^ 🡪 ln(t-tau) 🡪 ln(α-syn) 🡪 MoCA_4y_;

(Aβ_1-42_)^1/2^ 🡪 ln(α-syn) 🡪 MoCA_4y_;

(Aβ_1-42_)^1/2^ 🡪 RBD 🡪 UPDRS_1-3_ 🡪 MoCA_4y_;

(Aβ_1-42_)^1/2^ 🡪 RBD 🡪 UPDRS_1-3_ 🡪 MoCA_b_ 🡪 MoCA_4y_;

UPDRS_1-3_ 🡪 MoCA_b_ 🡪 MoCA_4y_;

RBD 🡪 UPDRS_1-3_ 🡪 MoCA_4y_;

RBD 🡪 UPDRS_1-3_ 🡪 MoCA_b_ 🡪 MoCA_4y_;

GDS 🡪 UPDRS_1-3_ 🡪 MoCA_4y_;

GDS 🡪 UPDRS_1-3_ 🡪 MoCA_b_ 🡪 MoCA_4y_;

GDS 🡪 RBD 🡪 UPDRS_1-3_ 🡪 MoCA_4y_;

GDS 🡪 RBD 🡪 UPDRS_1-3_ 🡪 MoCA_b_ 🡪 MoCA_4y_;

DaT 🡪 UPDRS_1-3_ 🡪 MoCA_4y_;

DaT 🡪 UPDRS_1-3_ 🡪 MoCA_b_ 🡪 MoCA_4y_;

DaT 🡪 RBD 🡪 UPDRS_1-3_ 🡪 MoCA_4y_;

DaT 🡪 RBD 🡪 UPDRS_1-3_ 🡪 MoCA_b_ 🡪 MoCA_4y_;

Education 🡪 MoCA_b_ 🡪 MoCA_4y_;

Gender 🡪 MoCA_b_ 🡪 MoCA_4y_.

The significant (*p* < 0.1) indirect effects on MoCA_b_ resulting from the first developed GSEM model (Figs. 1A,B) can be listed as follows:

Age 🡪 UPDRS_1-3_ 🡪 MoCA_b_;

Age 🡪 (Aβ_1-42_)^1/2^ 🡪 RBD 🡪 UPDRS_1-3_ 🡪 MoCA_b_;

(Aβ_1-42_)^1/2^ 🡪 RBD 🡪 UPDRS_1-3_ 🡪 MoCA_b_;

(p-tau)^-1/2^ 🡪 (Aβ_1-42_)^1/2^ 🡪 RBD 🡪 UPDRS_1-3_ 🡪 MoCA_b_;

DaT 🡪 UPDRS_1-3_ 🡪 MoCA_b_;

RBD 🡪 UPDRS_1-3_ 🡪 MoCA_b_;

DaT 🡪 RBD 🡪 UPDRS_1-3_ 🡪 MoCA_b_;

GDS 🡪 UPDRS_1-3_ 🡪 MoCA_b_;

GDS 🡪 RBD 🡪 UPDRS_1-3_ 🡪 MoCA_b_.

The significant (*p* < 0.1) indirect effects on MoCA_4y_ resulting from the second (alternative) developed GSEM model (Figs. 1A and 2) can be listed as follows:

Age 🡪 MoCA_b_ 🡪 MoCA_4y_;

Age 🡪 ln(t-tau) 🡪 MoCA_4y_;

Age 🡪 ln(t-tau) 🡪 ln(α-syn) 🡪 MoCA_4y_;

Age 🡪 UPDRS_1-3_ 🡪 MoCA_4y_;

Age 🡪 UPDRS_1-3_ 🡪 MoCA_b_ 🡪 MoCA_4y_;

(p-tau)^-1/2^ 🡪 ln(t-tau) 🡪 MoCA_4y_;

(p-tau)^-1/2^ 🡪 ln(t-tau) 🡪 ln(α-syn) 🡪 MoCA_4y_;

ln(t-tau) 🡪 ln(α-syn) 🡪 MoCA_4y_;

(Aβ_1-42_)^1/2^ 🡪 ln(t-tau) 🡪 MoCA_4y_;

(Aβ_1-42_)^1/2^ 🡪 ln(t-tau) 🡪 ln(α-syn) 🡪 MoCA_4y_;

(Aβ_1-42_)^1/2^ 🡪 ln(α-syn) 🡪 MoCA_4y_;

(Aβ_1-42_)^1/2^ 🡪 (p-tau)^-1/2^ 🡪 ln(t-tau) 🡪 MoCA_4y_;

(Aβ_1-42_)^1/2^ 🡪 (p-tau)^-1/2^ 🡪 ln(t-tau) 🡪 ln(α-syn) 🡪 MoCA_4y_;

(Aβ_1-42_)^1/2^ 🡪 RBD 🡪 UPDRS_1-3_ 🡪 MoCA_4y_;

(Aβ_1-42_)^1/2^ 🡪 RBD 🡪 UPDRS_1-3_ 🡪 MoCA_b_ 🡪 MoCA_4y_;

UPDRS_1-3_ 🡪 MoCA_b_ 🡪 MoCA_4y_;

RBD 🡪 UPDRS_1-3_ 🡪 MoCA_4y_;

RBD 🡪 UPDRS_1-3_ 🡪 MoCA_b_ 🡪 MoCA_4y_;

GDS 🡪 UPDRS_1-3_ 🡪 MoCA_4y_;

GDS 🡪 UPDRS_1-3_ 🡪 MoCA_b_ 🡪 MoCA_4y_;

GDS 🡪 RBD 🡪 UPDRS_1-3_ 🡪 MoCA_4y_;

GDS 🡪 RBD 🡪 UPDRS_1-3_ 🡪 MoCA_b_ 🡪 MoCA_4y_;

DaT 🡪 UPDRS_1-3_ 🡪 MoCA_4y_;

DaT 🡪 UPDRS_1-3_ 🡪 MoCA_b_ 🡪 MoCA_4y_;

DaT 🡪 RBD 🡪 UPDRS_1-3_ 🡪 MoCA_4y_;

DaT 🡪 RBD 🡪 UPDRS_1-3_ 🡪 MoCA_b_ 🡪 MoCA_4y_;

Education 🡪 MoCA_b_ 🡪 MoCA_4y_;

Gender 🡪 MoCA_b_ 🡪 MoCA_4y_.

The significant (*p* < 0.1) indirect effects on MoCA_b_ resulting from the second (alternative) developed GSEM model (Figs. 1A and 2) can be listed as follows:

Age 🡪 UPDRS_1-3_ 🡪 MoCA_b_;

(Aβ_1-42_)^1/2^ 🡪 RBD 🡪 UPDRS_1-3_ 🡪 MoCA_b_;

DaT 🡪 UPDRS_1-3_ 🡪 MoCA_b_;

RBD 🡪 UPDRS_1-3_ 🡪 MoCA_b_;

DaT 🡪 RBD 🡪 UPDRS_1-3_ 🡪 MoCA_b_;

GDS 🡪 UPDRS_1-3_ 🡪 MoCA_b_;

GDS 🡪 RBD 🡪 UPDRS_1-3_ 🡪 MoCA_b_.

# Validations of the GSEM models

The GSEM model developed in this study was based on simultaneous testing of multiple hypotheses resulting in multiple regressions constituting the final GSEM (Figs. 1A,B and 2). Therefore, the first validation step was based on the Bonferroni-type multiple comparison procedures including original Bonferroni approach [s4], Hochberg’s procedure [s5], and False Discovery Rate (FDR) controlling procedure [s6]. These three procedures determine whether or not regression coefficients in multiple regressions (with multiple predictor variables) are significant under an accepted threshold (of, for example, 0.05) for family-wise error rate [s6]. Thus, the indicated procedures allowed validation of significance of the obtained regression coefficients in the developed GSEM models (Figs. 1A,B and 2) in the case of possible mutual influences of multiple relationships in the models.

Each of the three procedures determines threshold values for the *p*-values in the developed models (Tables 1 and 2) under the adopted family-wise error rate. If the *p*-values in the models (Tables 1 and 2) are smaller than the threshold values determined by the procedures, then the respective regression coefficients are significant under the Bonferroni-like tests and the adopted family-wise error rate. If the *p*-values in the models (Figs. 1A,B and 2) are larger than the determined thresholds, then the respective regression coefficients are not significant.

**Table 3 in S1 Text. Validations for the GSEM model (Fig. 1A).**

| **Response Variable** | **Predictor Variable** | **Coefficient** | ***p*-value** | ***Hochberg’s*** | ***FDR*** |
| --- | --- | --- | --- | --- | --- |
| **MoCA_4y_** | MoCA_b_ | 0.549  (1.6%) | < 0.001  (< 0.001) | < 0.0040 | < 0.0042 |
|  | UPDRS_1-3_ | – 0.0497  (4.9%) | 0.002  (0.001) | < 0.015 | < 0.013 |
|  | Age | – 0.462  (1.2%) | 0.005  (0.003) | < 0.022 | < 0.017 |
|  | GDS | – 0.202  (1.4%) | 0.031  (0.043) | < 0.27 | < 0.046 |
|  | ln(α-syn) | – 332.49  (4.8%) | 0.027  (0.046) | < 0.11 | < 0.038 |
|  | ln^2^(α-syn) | 45.172  (4.5%) | 0.026  (0.043) | < 0.058 | < 0.029 |
|  | ln^3^(α-syn) | – 2.0306  (2.2%) | 0.026  (0.042) | < 0.080 | < 0.033 |
|  | ln(t-tau) | – 2.769  (1.5%) | 0.001  (0.027) | < 0.0090 | < 0.0083 |
|  | (Aβ_1-42_)^1/2^ | 12.010  (0.89%) | 0.020  (0.052) | < 0.031 | < 0.021 |
|  | Aβ_1-42_ | – 0.60407  (0.46%) | 0.028  (0.057) | < 0.17 | < 0.042 |
|  | (Aβ_1-42_)^3/2^ | 0.01015  (0.011%) | 0.034  (0.060) | < 0.60 | < 0.050 |
|  | (Aβ_1-42_)^1/2^ × Age | 0.0192  (1.9%) | 0.020  (0.016) | < 0.043 | < 0.025 |
| **MoCA_b_** | UPDRS_1-3_ | – 0.0248  (1.2%) | 0.038  (0.041) | < 0.067 | ***< 0.033*** |
|  | Education | 0.1393  (1.6%) | 0.011  (0.014) | < 0.020 | < 0.017 |
|  | Age | – 0.0572  (0.073%) | 0.043  (0.037) | < 0.13 | *< 0.042* |
|  | Age^2^ | 0.00808  (2.2%) | 0.017  (0.016) | < 0.038 | < 0.025 |
|  | Age^3^ | - | 0.74  (0.73) | - | - |
|  | Age^4^ | – 0.000018  (2.8%) | 0.006  (0.005) | < 0.0083 | < 0.0083 |
|  | Gender (base: Male) | 0.631  (0.052%) | 0.063  (0.062) | < 0.30 | ***< 0.050*** |
| **UPDRS_1-3_** | RBD | 1.2098  (0.67%) | < 0.001  (< 0.001) | < 0.013 | < 0.013 |
|  | DaT | – 8.932  (1.3%) | < 0.001  (< 0.001) | < 0.033 | < 0.025 |
|  | GDS | 1.230  (1.5%) | < 0.001  (0.003) | < 0.075 | < 0.038 |
|  | Age | 0.1655  (0.77%) | 0.031  (0.035) | < 0.20 | < 0.050 |
| **RBD** | (Aβ_1-42_)^1/2^ | – 0.173  (0.35%) | 0.005  (0.005) | < 0.017 | < 0.017 |
|  | DaT | – 1.03  (1.2%) | 0.016  (0.027) | < 0.15 | < 0.050 |
|  | GDS | 0.169  (0.25%) | 0.015  (0.029) | < 0.050 | < 0.033 |

The outcomes of validations of the GSEM model in Fig. 1A, using the bootstrapping approach with 500 bootstrapping re-samplings and Hochberg’s and FDR procedures for simultaneous testing of multiple hypotheses under the family-wise error rate of 0.05. The third and fourth columns present the regression coefficients and their respective *p*-values from the GSEM model (Fig. 1A and Table 1), with the bootstrapping *p*-values and percentage biases for the regression coefficients given in brackets. The two rightmost columns show the *p*-value thresholds for the Hochberg’s and FDR procedures. The determined model *p*-values (from the column ‘*p*-value’) should be smaller than the shown respective thresholds.

**Table 4 in S1 Text. Validations for the p-tau GSEM Network in Fig. 1B (p-tau Model).**

| **Response Variable** | **Predictor Variable** | **Coefficient** | ***p*-value** | ***Hochberg’s*** | ***FDR*** |
| --- | --- | --- | --- | --- | --- |
| **ln(α-syn)** | (Aβ_1-42_)^1/2^ | 0.0219  (0.067%) | 0.006  (0.003) | < 0.025 | < 0.025 |
|  | ln(t-tau) | 0.6773  (0.36%) | < 0.001  (< 0.001) | < 0.10 | < 0.050 |
| **(Aβ_1-42_)^1/2^** | (p-tau)^-1/2^ | – 16.91  (1.2%) | < 0.001  (< 0.001) | < 0.025 | < 0.025 |
|  | Age | – 0.0361  (1.2%) | 0.017  (0.013) | < 0.10 | < 0.050 |
| **ln(t-tau)** | (p-tau)^-1/2^ | 3.0912  (0.81%) | 0.076  (0.059) | < 0.35 | ***< 0.050*** |
|  | (p-tau)^-1^ | – 9.114  (0.43%) | 0.003  (0.001) | < 0.083 | < 0.036 |
|  | (Aβ_1-42_)^1/2^ | 0.0453  (0.26%) | < 0.001  (< 0.001) | < 0.0071 | < 0.0071 |
|  | Age | 0.0923  (1.1%) | < 0.001  (0.001) | < 0.017 | < 0.014 |
|  | Age^2^ | 0.0002936  (7.8%) | 0.031  (0.034) | < 0.15 | < 0.043 |
|  | (p-tau)^-1/2^ × Age | – 0.1014  (3.3%) | 0.002  (0.005) | < 0.050 | < 0.029 |
|  | (Aβ_1-42_)^1/2^ × Age | – 0.00275  (0.30%) | 0.001  (0.006) | < 0.030 | < 0.021 |

The outcomes of validations of the p-tau Model in Fig. 1B (Table 2), using the bootstrapping approach with 500 bootstrapping re-samplings and Hochberg’s and FDR procedures for simultaneous testing of multiple hypotheses under the family-wise error rate of 0.05. The third and fourth columns present the regression coefficients and their respective *p*-values from the p-tau model (Fig. 1B and Table 2), with the bootstrapping *p*-values and percentage biases for the regression coefficients given in brackets. The two rightmost columns show the *p*-value thresholds for the Hochberg’s and FDR procedures. The determined model *p*-values (from the column ‘*p*-value’) should be smaller than the shown respective thresholds.

**Table 5 in S1 Text. Validations for the (Aβ_1-42_)^1/2^ GSEM Network in Fig. 2 (Amyloid Model).**

| **Response Variable** | **Predictor Variable** | **Coefficient** | ***p*-value** | ***Hochberg’s*** | ***FDR*** |
| --- | --- | --- | --- | --- | --- |
| **ln(α-syn)** | (Aβ_1-42_)^1/2^ | 0.0219  (0.36%) | 0.006  (0.007) | < 0.10 | < 0.050 |
|  | ln(t-tau) | 0.6773  (0.28%) | < 0.001  (< 0.001) | < 0.025 | < 0.025 |
| **(p-tau)^-1/2^** | (Aβ_1-42_)^1/2^ | 0.0443  (1.3%) | 0.001  (0.003) | < 0.025 | < 0.025 |
|  | Aβ_1-42_ | – 0.00146  (1.2%) | < 0.001  (< 0.001) | < 0.10 | < 0.050 |
| **ln(t-tau)** | (p-tau)^-1/2^ | – 2.068  (0.16%) | < 0.001  (< 0.001) | < 0.0071 | < 0.0071 |
|  | (Aβ_1-42_)^1/2^ | 0.192  (0.38%) | 0.006  (0.015) | < 0.083 | < 0.036 |
|  | Aβ_1-42_ | – 0.00383  (0.92%) | 0.034  (0.051) | < 0.35 | < 0.050 |
|  | Age | 0.0990  (1.7%) | < 0.001  (< 0.001) | < 0.017 | < 0.014 |
|  | Age^2^ | 0.000324  (3.8%) | 0.019  (0.020) | < 0.15 | < 0.043 |
|  | (p-tau)^-1/2^ × Age | – 0.0923  (0.16%) | 0.005  (0.007) | < 0.050 | < 0.029 |
|  | (Aβ_1-42_)^1/2^ × Age | – 0.00318  (2.9%) | < 0.001  (0.003) | < 0.030 | < 0.021 |

The outcomes of validations of the Amyloid Model in Fig. 2 (Table 2), using the bootstrapping approach with 500 bootstrapping re-samplings and Hochberg’s and FDR procedures for simultaneous testing of multiple hypotheses under the family-wise error rate of 0.05. The third and fourth columns present the regression coefficients and their respective *p*-values from the Amyloid Model (Fig. 2 and Table 2), with the bootstrapping *p*-values and percentage biases for the regression coefficients given in brackets. The two rightmost columns show the *p*-value thresholds for the Hochberg’s and FDR procedures. The determined model *p*-values (from the column ‘*p*-value’) should be smaller than the shown respective thresholds.

The original Bonferroni approach is typically excessively conservative, that is, introduces too strict and unjustified limitations on the validity of regression coefficients in multiple regression models [s6]. Accordingly, it is not normally used in the modern multiple comparison analyses [s6]. The application of the original Bonferroni approach to the developed GSEM models (Figs. 1A,B and 2) expectedly resulted in some significance failures for some of the regression coefficients. However, it is important to understand that these failures do not constitute lack of validation for the developed models due to the excessively conservative nature of the original Bonferroni approach [s6]. Therefore, we do not present the outcomes of the Bonferroni approach in this study.

Validation of the developed GSEM models was conducted using the more contemporary and more reasonable approaches based on the Hochberg’s procedure [s5] and False Discovery Rate (FDR) controlling procedure [s6]. The validation outcomes resulting from these two procedures for the models shown in Figs. 1A,B and 2 are presented in Tables 3, 4 and 5 in S1 Text. These outcomes are given for the family-wise error rate of 0.05. The two rightmost columns in Tables 3, 4 and 5 in S1 Text show the thresholds for *p*-values, calculated from the Hochberg’s and FDR procedures. According to these validation procedures, for the regression coefficients to be significant in the GSEM model shown in Figs. 1A,B and 2, the *p*-values in the fourth columns in Tables 3, 4 and 5 in S1 Text should be smaller than the respective Hochberg’s and FDR thresholds (which is why the ‘<’ signs in the columns with the thresholds). In particular, it can be seen that all the regression coefficients are significant under the Hochberg’s procedure (Tables 3, 4 and 5 in S1 Text). Only three coefficients failed the FDR procedure (marked by Italic Bold font in Tables 3 and 4 in S1 Text). However, if the family-wise error rate of 0.1 is adopted instead of 0.05, then all of the regression coefficients in the developed models are also significant under the FDR procedure.

It can be concluded from here that the developed models and their regression coefficients were validated under the Hochberg’s and FDR procedures and the family-wise error rate of 0.05. The only possible exceptions in the FDR procedure were the three regression coefficients that were further validated under the family-wise error rate of 0.1 (under 10% significance). Therefore, the available data sample is sufficient to justify the conclusions of the study.

Internal cross-validations of the developed GSEM models (Figs. 1A,B and 2) were conducted using the bootstrapping procedure with 500 re-samplings [49,s7]. For MoCA scores as dependent variables, the bootstrapping procedure was conducted using the Tobit regression [50] because of the censored nature of the MoCA data (see also Figure 1 in S1 Text).

The outcomes of the bootstrapping procedure are also presented in Tables 3, 4 and 5 in S1 Text. For the regression coefficients, the percentages in brackets indicate the bootstrapping biases of these coefficients, and the bootstrapping *p*-values are given in brackets in the ‘*p*-value’ columns (Tables 3, 4 and 5 in S1 Text).

The bootstrapping percentage biases for all regression coefficients are no greater than in single digits and demonstrate good agreement with the regression coefficients in the developed models (Tables 3, 4 and 5 in S1 Text). None of the bootstrapping *p*-values demonstrate any breaches of statistical significance of the respective coefficients beyond the 0.1 significance threshold. It could thus be concluded that the bootstrapping technique further validated the GSEM models developed in this study (Figs. 1A,B and 2 and Tables 1 and 2).

# Supporting References in S1 Text

1. Johnson JB, Omland K. Model section in ecology and evolution. Trends in Ecol. and Evolution. 2004; 19: 101–108.
2. Bobela W, Aebischer P, Schneider BL. Alpha-Synuclein as a mediator in the interplay between aging and Parkinson’s disease. Biomolecules. 2015;5: 2675-2700.
3. Mikolaenko I, Pletnikova O, Kawas CH, O’Brien R, Resnick SM, Crain B, et al. Alpha-synuclein lesions in normal aging, Parkinson disease, and Alzheimer disease: Evidence from the Baltimore Longitudinal Study of Aging (BLSA). J. Neuropathol. Exp. Neurol. 2005;64(2): 156-162.
4. Bland JM, Altman DG. Multiple significance tests: the Bonferroni method. BMJ. 1995;310: 170.
5. Hochberg Y. A sharper Bonferroni procedure for multiple tests of significance. Biometrika. 1988;75: 800-802.
6. Benjamini Y, Hochberg Y. Controlling the false discovery rate: a practical and powerful approach to multiple testing. J. R. Stat. Soc. Series B. 1995;57: 289-300.
7. Mooney CZ, Duval RD. Bootstrapping: A Nonparametric Approach to Statistical Inference. Sage, Newbury Park, CA; 1993.
